# CMRLCCOA: Multi-Strategy Enhanced Coati Optimization Algorithm for Engineering Designs and Hypersonic Vehicle Path Planning

**DOI:** 10.3390/biomimetics9070399

**Published:** 2024-07-01

**Authors:** Gang Hu, Haonan Zhang, Ni Xie, Abdelazim G. Hussien

**Affiliations:** 1 Department of Applied Mathematics, Xi’an University of Technology, Xi’an 710054, China; zhanghaonan2023@163.com (H.Z.); xieni2024@sina.com (N.X.); 2 School of Computer Science and Engineering, Xi’an University of Technology, Xi’an 710048, China; 3 Department of Computer and Information Science, Linköping University, 58183 Linköping, Sweden; abdelazim.hussien@liu.se; 4 Faculty of Science, Fayoum University, Faiyum 63514, Egypt; 5 Applied Science Research Center, Applied Science Private University, Amman 11931, Jordan; 6 MEU Research Unit, Middle East University, Amman 11831, Jordan

**Keywords:** coati optimization algorithm, chaos mapping strategy, Lévy flight strategy, lens imaging reverse learning strategy, crossover strategy

## Abstract

The recently introduced coati optimization algorithm suffers from drawbacks such as slow search velocity and weak optimization precision. An enhanced coati optimization algorithm called CMRLCCOA is proposed. Firstly, the Sine chaotic mapping function is used to initialize the CMRLCCOA as a way to obtain better-quality coati populations and increase the diversity of the population. Secondly, the generated candidate solutions are updated again using the convex lens imaging reverse learning strategy to expand the search range. Thirdly, the Lévy flight strategy increases the search step size, expands the search range, and avoids the phenomenon of convergence too early. Finally, utilizing the crossover strategy can effectively reduce the search blind spots, making the search particles constantly close to the global optimum solution. The four strategies work together to enhance the efficiency of COA and to boost the precision and steadiness. The performance of CMRLCCOA is evaluated on CEC2017 and CEC2019. The superiority of CMRLCCOA is comprehensively demonstrated by comparing the output of CMRLCCOA with the previously submitted algorithms. Besides the results of iterative convergence curves, boxplots and a nonparametric statistical analysis illustrate that the CMRLCCOA is competitive, significantly improves the convergence accuracy, and well avoids local optimal solutions. Finally, the performance and usefulness of CMRLCCOA are proven through three engineering application problems. A mathematical model of the hypersonic vehicle cruise trajectory optimization problem is developed. The result of CMRLCCOA is less than other comparative algorithms and the shortest path length for this problem is obtained.

## 1. Introduction

An optimization problem is to achieve the optimal value of the design objective under certain constraints. Optimization problems exist widely in intelligent production, engineering manufacturing, agricultural development, and many other fields [1]. But as the rapidly evolving digital age, data are showing explosive growth, and there are more and more multidimensional and multimodal problems, making many real-world problems more complex and diverse [2]. For traditional mathematical optimization means, such as the gradient descent method [3], conjugate gradient method [4], and quasi-Newton method [5], they often have some limitations when handling both discrete and other questions [6]. They have a tendency to trap into local optimal solutions, slow convergence speed, or low computational accuracy. Therefore, it is very difficult to use traditional algorithms for calculation and extracting meaningful information. Generally speaking, solving NP hard problems involves finding a point in a multidimensional hyperspace, which is the optimal solution. However, the identification process is very complex, time-consuming, and computationally expensive. Therefore, it is important to find a biomimetic computing method that is fast and effective [1].

Nowadays, metaheuristic algorithms have been proven to be competitive alternative algorithms, often used to solve highly complicated nonlinear optimization issues, such as multi-objective optimization problems [7], multimodal optimization problems [8], and complex constraint optimization problems [9].

Metaheuristic algorithms can avoid local optima and have faster convergence speed, better robustness, and higher stability than traditional algorithms [10]. This field has been developed so far that quite a number of very classical algorithms have been proposed. This mainly includes a genetic algorithm (GA) that emulates the evolutionary processes of living species [11], differential evolution (DE) that optimizes a search through cooperation and competition among individuals within a group [12], artificial immune systems (AIS) for modeling the body immune response mechanism [13], an ant colony algorithm (ACO) using ants’ way finding behavior as a model [14], particle swarm optimization (PSO) modeling the actions of birds in search of food [15], a simulated annealing algorithm (SA) modeled on the annealing procedure with solid materials [16], and a taboo search algorithm (TSA) for modeling the human intellectual memory procedure [17]. They always emerge from the imitation or exposition of specific natural occurrences and sequences, or the cognitive actions of living collectives and have the characteristics of simplicity, universality, and ease of parallel processing.

Based on sources of inspiration, metaheuristic algorithms mainly include evolution algorithms, human behavior-based algorithms, physics and chemistry-based algorithms, and swarm intelligence-based algorithms [18,19].

The evolutionary algorithm is based on concepts such as biology and genetics and is built by modeling the laws of nature’s superiority and inferiority. They achieve population progress according to the laws of natural selection, and thus finish the best solution. Conventional evolutionary algorithms are primarily represented by GA and DE. Both algorithms are modeled from the principle of reproduction in nature and then use strategies such as crossover, selection, and mutation to update the population.

Human behavior-based algorithms are inspired by human performance, such as self-learning actions and social activities [20]. The most commonly used algorithm is the imperialist competition algorithm [21], social-based algorithms [22], league championship algorithm [23], and poor and rich optimization algorithm [24].

The algorithms based on physics and chemistry mainly come from the physical laws and chemical phenomena in the universe. Among them, SA mentioned above is a classical algorithm. Furthermore, there are many algorithms developed from physical laws, such as gravity search algorithms [25] based on the law of universal gravitation; chaos optimization algorithms [26] based on the traversal, randomness, and regularity of chaotic phenomena; optical optimization algorithms [27] based on the principle of optical reflection; and black hole algorithms based on strong attractive forces [28].

Swarm intelligence-based algorithms simulate the behavior of natural populations such as ants, birds, bees, whales, lions, wolves, etc. Each population is a population of organisms. Groups search for the best location among themselves through behaviors such as cooperation and hunting. The representative algorithms are PSO and ACO referred to above. In addition, there are many algorithms of this type, such as beluga whale optimization [29], grey wolf optimizer [30], marine predator algorithm [31], white shark optimizer [32], emperor penguin optimizer [33], and so on.

For metaheuristic algorithms, the ability to explore and develop directly determines the performance of the algorithms [34]. Their imbalance will directly cause a reduction in the precision of problem-solving. Weak exploration ability will affect the population to explore more places, which will lead to getting trapped at local optima. And the lack of exploitation ability will directly affect the population’s ability to find the optimal value. This is likewise a prevalent issue with current optimization methodologies. At the same time, this is exactly what is meant by improvements to the algorithm.

M. Dehghani et al. [35] presented the coati optimization algorithm (COA) in 2023. Coatis are very active, agile in movement, and have strong adaptability. They forage during the day and rest on trees at night. Iguanas are one of the favorite foods of long coatis, and coatis often cooperate to prey on them. In addition, long coatis also face the risk of being preyed upon. Thus, COA was inspired by the strategies used by long-haired coatis when they are attacking iguanas, as well as the strategies they use when facing and avoiding predators. Although COA shows a high level of competitiveness on some of the problems, it still has room for improvement. According to the literature [36], COA always exhibits a state of premature convergence and is highly susceptible to falling into a local optimum. Meanwhile, during the experiments, the performance of COA relative to some newly proposed superior metaheuristic algorithms is always at a disadvantage when facing large-scale problems. And the disadvantage of low diversity in COA populations cannot be ignored either. As a result, many researchers have enhanced COA to solve more sophisticated engineering problems. F.A. Hashim et al. [37] proposed an efficient adaptive mutation COA and applied it to feature selection and global optimization. P. Tamilarasu and G. Singaravel [38] use an improved COA to achieve efficient scheduling in cloud computing environments. K. Thirumoorthy and J.J.B. J [39] improved the COA and applied it to breast cancer classification.

Nevertheless, the No Free Lunch derivation [40] has indicated that no single algorithm is capable of addressing every optimization challenge flawlessly. Excellent performance on one problem may not lead to a viable solution on another unrelated problem. As a result, researchers need to constantly develop new algorithms or make targeted improvements to certain algorithms to cope with increasingly complex real-world problems. Therefore, the improvement in some existing algorithms is very necessary.

Consequently, in this paper, the chaotic mapping strategy, lens imaging reverse learning strategy, crossover strategy, and Lévy flight strategy are applied to improve the COA. Firstly, the chaotic mapping strategy [1] is introduced in the population initialization stage to use chaotic sequences for population initialization to obtain higher-quality populations. Secondly, the use of the lens imaging reverse learning strategy [41] not only improves population diversity but also enlarges the scope of the search. In the early stage, the Lévy flight strategy [42] is applied. It allows the population to get rid of partial optima and expand the search capability. In the end, the introduction of the crisscross optimization algorithm [43] helps to amend the phenomenon of early convergence of the algorithm. The amalgamation of these strategies augments the optimization capability of the COA. The innovations as well as the main contributions of this paper are as follows.

(1) The enhanced COA consists of four strategies, namely the chaotic mapping strategy, the lens imaging reverse learning strategy, the Lévy flight strategy, and the crossover strategy.
(2) The effect of 10 common chaotic strategies to improve the COA is analyzed and the optimal strategy is finally selected.
(3) The CMRLCCOA is compared with the primitive COA, six new algorithms proposed in the last two years, four classic and well-recognized algorithms, and three improved algorithms, which are tested with the functions included in the CEC2017 and CEC2019 function sets. In addition, *dim* = 50 and 100 were also selected in the CEC2017 test set.
(4) CMRLCCOA is used to solve three engineering optimization problems, including a single-stage cylindrical gear reducer, a welded beam design problem, and a cantilever beam design problem.
(5) This paper establishes a mathematical model of the cruise trajectory of a hypersonic vehicle and solves the path planning problem with the newly proposed CMRLCCOA. Furthermore, the results of nine algorithms are compared. Thus, the reliability of CMRLCCOA is verified.

The remainder of this paper is organized as follows: Section 2 briefly describes the mathematical model of COA. Section 3 describes the detailed structure of the CMRLCCOA algorithm. The performance of the CMRLCCOA is evaluated on the basis of numerical experimental results in Section 4. Section 5 solves three real-world problems using CMRLCCOA. In Section 6, The cruise ballistic trajectory problem for hypersonic vehicles is modeled and solved using CMRLCCOA. Finally, Section 7 summarizes this paper.

## 2. Introduction to Mathematical Modeling of Coati Optimization Algorithm

The coati optimization algorithm (COA) is a new metaheuristic algorithm proposed in 2023 [35]. It is inspired by two natural behaviors of the coatis, including strategies for cooperating in attacking iguanas and behavioral strategies for facing and escaping predators. In p-dimensional space, each coati acts as a separate searching individual. The hunting process and escape process of the coati from predators are both individual updates. The position of the coati will be dynamically adjusted according to the position of the iguana and the migrated locations, and finally the globally optimal location (the best candidate solution) will be selected. Next, we will briefly introduce the mathematical model of COA.

### 2.1. Initialization Process

Firstly, the COA initializes m random individuals, $x_{1},\ldots ,x_{i},\ldots x_{m}$, by Equation (1), where the maximum boundary for individual values is $X_{\max}=(x_{1}^{\max},\ldots ,x_{p}^{\max})$ and the minimum boundary is $X_{\min}=(x_{1}^{\min},\ldots ,x_{p}^{\min})$. Then, evaluate the initialized random individuals through the objective function.

(1) $$x_{i,j}=x_{j}^{\min}+rand\cdot (x_{j}^{\max}-x_{j}^{\min}),\;i=1,2,\ldots m,\;j=1,2,\ldots ,p.$$

### 2.2. Hunting and Attack Strategies (Exploration Phase)

**Figure 1:**
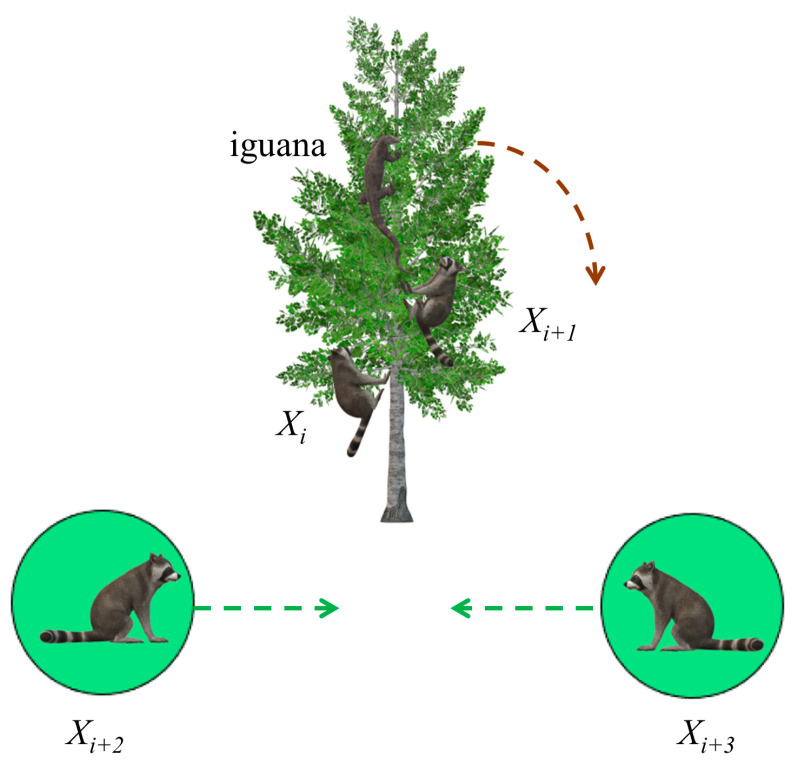
Coatis attacking iguana.

Coatis attack iguanas in groups. In this strategy, the coatis are first divided into two groups. One group climbs up a tree to approach and scare the iguana, while the other group waits quietly on the ground. When the iguana drops, the raccoons quickly attack and catch it. Figure 1 shows coatis attacking an iguana.

There are two assumptions in this strategy. First, the iguana is assumed to be in the optimal position. Second, half of the species climbed trees and half waited on the ground. Equation (2) is used to simulate the process of coatis climbing trees.

(2) $$x_{i,j}^{new}=x_{i,j}+b\cdot (Igu_{j}-I\cdot x_{i,j}),\;i=1,2,\ldots ,\left\lfloor \frac{m}{2}\right\rfloor .$$

**Figure 2:**
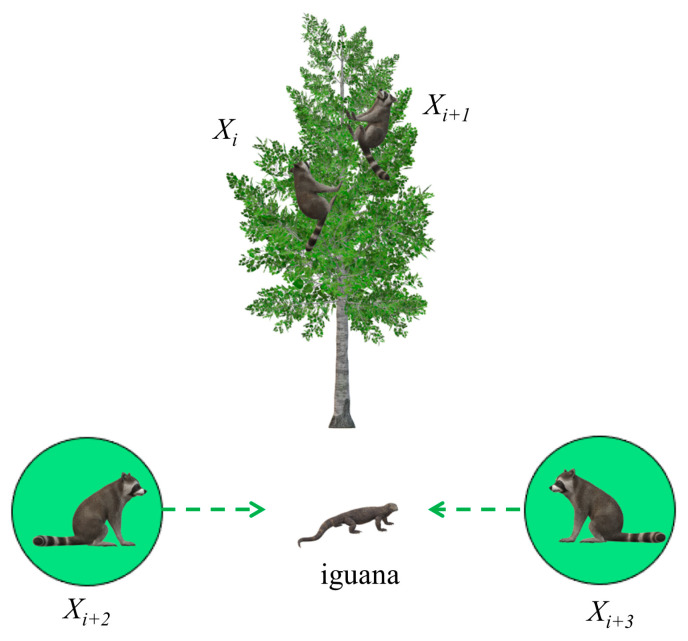
Iguana update location.

When the iguana is frightened to land, the position of the iguana is set randomly; however, the other half of the coatis waiting on the ground move according to the random placement of the iguana. This behavior is represented by Equations (3) and (4). A schematic of the iguana’s updated position is shown in Figure 2.

(3) $$Igu_{j}=x_{j}^{\min}+b\cdot (x_{j}^{\max}-x_{j}^{\min}),\;j=1,2,\ldots ,p,$$

(4) $$x_{i,j}^{new}=\left\{\begin{array}{l} x_{i,j}+b\cdot (Igu_{j}-I\cdot x_{i,j}),\;F_{Igu}<F_{i}\\ x_{i,j}+b\cdot (x_{i,j}-Igu_{j}),\;F_{Igu}>F_{i} \end{array}\right.,\;i=\left[\frac{N}{2}\right]+1,\left[\frac{N}{2}\right]+2,\ldots ,N,\;j=1,2,\ldots ,m,$$

where $x_{i,j}$ represents the *j*-th dimension of the *i*-th coati, and *b* is a stochastic number between [0, 1]. *Igu* is the randomly given location of the iguana. *I* is any stochastic value in 1 and 2.

### 2.3. The Stage of Escaping Predators (Exploitation Stage)

**Figure 3:**
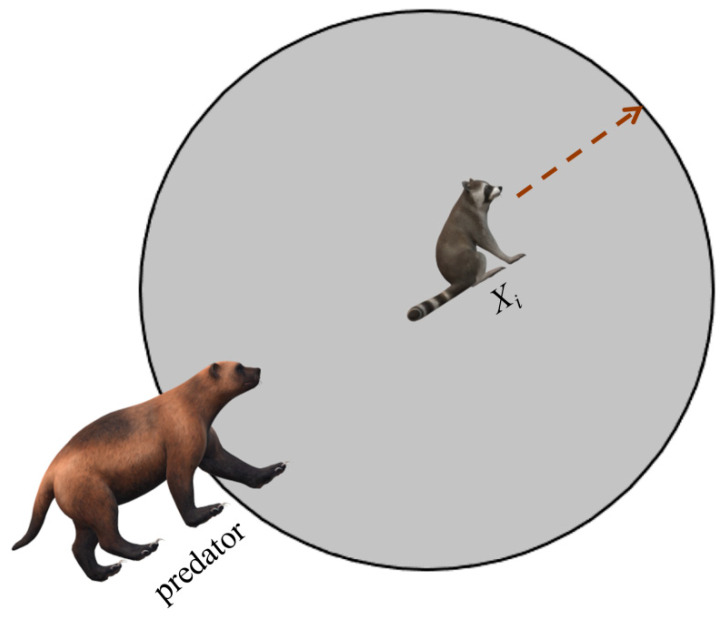
A schematic illustration of the behavior of a coati fleeing from a predator.

During the development phase, the strategy adopted by the coati in facing and escaping predators is used to update its position. When a predator captures a coati, the coati quickly runs away and enters a relatively safe position, approaching the optimal position, as shown in Figure 3. This strategy demonstrates the capability of the COA algorithm development.

The strategy during the development phase is simulated using Equations (5) and (6).

(5) $$low_{j}=\frac{x_{j}^{\min}}{t},\;up_{j}=\frac{x_{j}^{\max}}{t},t=1,2,...,T,$$

(6) $$x_{i,j}^{new}=x_{i,j}+(1-2b)\cdot (low_{j}+b\cdot (up_{j}-low_{j})),\;i=1,2,...,m;\;j=1,2,...,p$$

where *low_j_* and *up_j_* indicate the local lower and upper bound of the *j*-th dimension, a stochastic number with a value of b between [0, 1].

If the updated adaptation value for the coati is preferable to the original adaptation value, the value is accepted; otherwise, do not accept this value. Equation (7) represents the update process.

(7) $$X_{i}=\left\{\begin{array}{l} X_{i}^{new},\;F_{i}^{new}<F_{i}\\ X_{i},\;\mathrm{else} \end{array}\right..$$

## 3. Multi-Strategy Enhanced COA

This part uses four strategies to strengthen COA. These strategies are the chaotic mapping strategy, lens imaging reverse learning strategy, Lévy flight strategy, and crossover strategy. The newly proposed CMRLCCOA solves the shortcomings of COA, which is prone to local optimization and premature convergence. The improvement strategy of the algorithm is presented next and the results are briefly analyzed.

### 3.1. Chaos Mapping Strategy

The traditional COA adopts the method of randomly setting the initial population, which is difficult to spread throughout the population, resulting in a lack of diversity in the original coati population and restricting the flexibility. Chaos mapping was first proposed by Lorenz et al. in 1963 [44]. Chaos mapping has characteristics such as randomness, traversal, and regularity [45]. This strategy can guarantee the diversity of the original population. Therefore, many intelligent algorithms employ chaotic mapping strategies to strengthen the optimization of algorithms. Zeng et al. use chaotic mapping to generate a random and regular initialization particle swarm, improving global search capability [46]. Xin et al. applied the chaotic mapping method for reinforcing the sparrow optimization algorithm [47].

**Figure 4:**
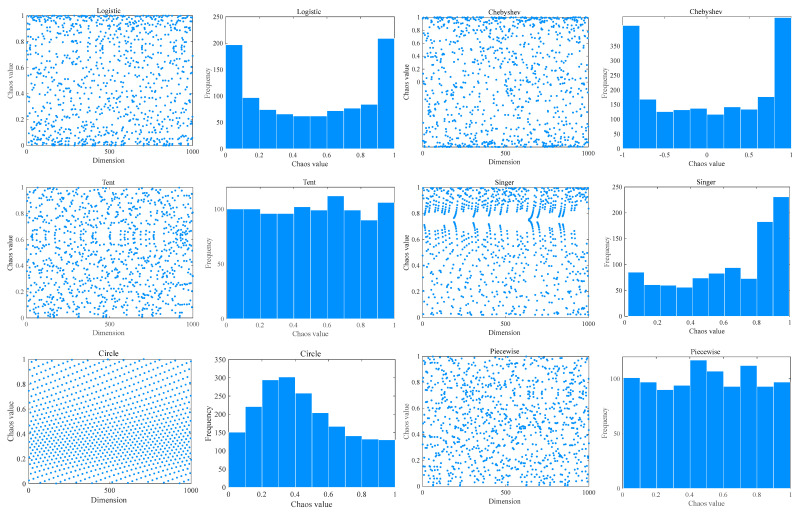
The schematics of chaotic mapping functions.

Chaos theory mainly studies the behavior of dynamic systems that are sensitive to initial states. The method of generating an initial population through chaotic mapping is to first use a one-dimensional chaotic map, specify a random initial value in it, and iteratively generate a series of continuous points. Chaotic mapping strategies can boost the competence of population diversity, success rate, and convergence. Table 1 describes ten common chaotic mapping functions. To have a clearer perception of these functions, Figure 4 visualizes some of the initialization functions. The image shows that these mappings allow random initial population positions to be evenly distributed in the search space. For the selection of different initialization methods, please see Section 4.3.

### 3.2. Reverse Learning Strategy for Lens Imaging

Many intelligent optimization algorithms have low population diversity in the later stages of the iteration and do not easily search for optimal solutions. It is difficult to jump out when a coati searching for an individual falls into a local optimum. Consequently, a specular reflection learning strategy is introduced in this paper. This strategy is an optimization mechanism [58], which extends the algorithm’s search area by computing the inverse solution at the current position. Therefore, it increases the likelihood of discovering the ideal solution. However, reverse learning strategies need to be combined with the principle of lens imaging to achieve better results [59].

**Figure 5:**
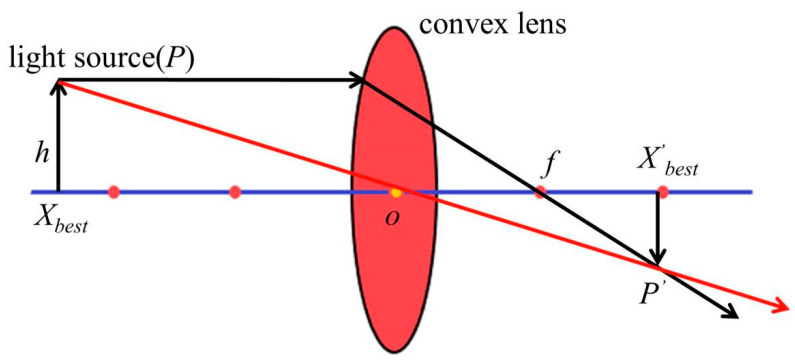
A schematic diagram of the principle of convex lens imaging.

Imaging by a convex lens is an optical law. A convection lens has an object and a solid image on each side of the lens. The diagram is depicted in Figure 5.

The lens imaging formula can be derived from Figure 5 as follows:

(8) $$\frac{1}{u}+\frac{1}{v}=\frac{1}{f}$$

where *u* is the object distance, *v* is the distance imaged, and *f* is the focal length.

**Figure 6:**
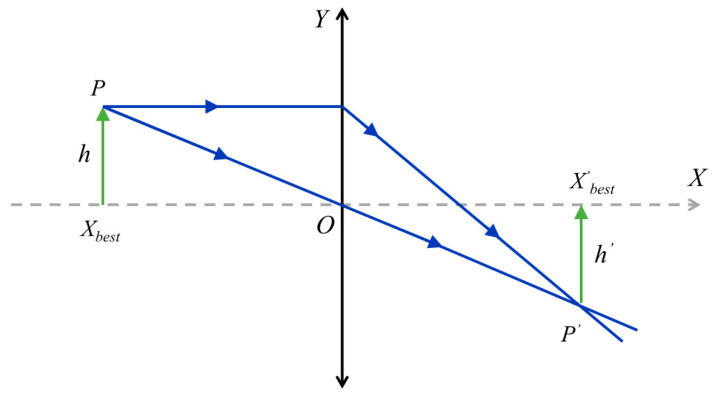
Schematic diagram of reverse learning strategy for convex lens imaging.

For the reverse learning strategy of convex lens imaging, an individual *P* is imaged in a convex lens as in one-dimensional space. This is shown in Figure 6. The principle of lens imaging is expressed as Equation (9).

(9) $$X^{\prime }=\frac{p+q}{2}+\frac{p+q}{2k}-\frac{X}{k}.$$

Equation (10) is the solution formula for reverse learning of lens imaging, which is extended to *D*-dimensional optimization problems. The reverse learning formula based on lens imaging is obtained as follows:

(10) $${X^{\prime }}_{j}=\frac{p_{j}+q_{j}}{2}+\frac{p_{j}+q_{j}}{2k}-\frac{X_{j}}{k}.$$

Among them, *p_j_* is the minimum in the *j*-th dimension, and *q_j_* is the maximum in the *j*-th dimension. *X_j_’* and *X_j_* are the inverse solutions of the lens.

### 3.3. Lévy Flight Strategy

**Figure 7:**
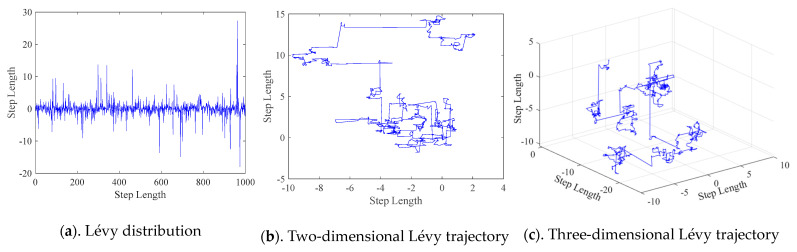
The Lévy flight trajectory.

In the COA, the position update is highly influenced by the iguana. However, the position update range of iguanas is small, so the search space and solution space of this algorithm are limited. The Lévy flight strategy is a stochastic behavior strategy proposed by Paul Lévy in 1937 [60], used to simulate the step size and direction during random walking or search processes. In this paper, the Lévy flight strategy is incorporated into the search phase of the COA to enlarge the search scope. Figure 7 depicts the Lévy distribution along with their trajectories in two- and three-dimensional spaces. This random wandering behavior can be effective in increasing the diversity of populations, which in turn allows individuals to explore a wider range of space. Then, the Lévy flight process can be described as a random walk process, as shown in Equation (11).

(11) $$\mathrm{Levy}(\lambda )\sim u=t^{-1-\lambda },\;0<\lambda \leq 2.$$

The *λ* can be calculated using the Mantegna method, as shown in Equation (12).

(12) $$s=\frac{\mu }{{\left|v\right|}^{\frac{1}{\lambda }}},$$

where *λ* is set to be 1.5, and *μ* and *v* follow a normal distribution.

(13) $$\sigma_{v}=1,$$

(14) $$\sigma_{\mu }={\left[\frac{\Gamma (1+\lambda )\cdot \sin(\pi \lambda /2)}{\lambda \cdot \Gamma \left[(1+\lambda )/2\right]\cdot 2^{(\lambda -1)/2}}\right]}^{\frac{1}{\beta }},$$

where Γ is the gamma function.

Therefore, Equation (15) is utilized to change the position of the coati.

(15) $${\bar{x}}_{t+1}={\bar{x}}_{t}^{rand}+\alpha \cdot \mathrm{sign}(rand-1/2)⊕\mathrm{Levy}(\lambda ),$$

where sign(*rand* – ½) can take three values, namely −1, 0, or 1. *α* represents the control quantity of step length, which can be expressed using Equation (16).

(16) $$\alpha =\alpha_{0}\left\lfloor {\bar{x}}_{t}^{rand}-{\bar{x}}_{t}\right\rfloor ,$$

where *α_0_* is set to be 0.01.

Then, Equation (15) can be represented as

(17) $${\bar{x}}_{t+1}={\bar{x}}_{t}^{rand}+\alpha_{0}\cdot \mathrm{sign}(rand-0.5)\cdot \frac{\mu }{{\left|v\right|}^{\frac{1}{\lambda }}}\cdot ({\bar{x}}_{t}^{rand}-{\bar{x}}_{t}).$$

### 3.4. Cross Optimization Algorithm

Meng et al. proposed the crisscross optimization algorithm (CSO) [43]. The algorithm utilizes horizontal and vertical crossing to update information, which can effectively solve the local optimization problem.

#### 3.4.1. Horizontal Crossover

Before performing the crossover operation, two individuals are paired. Subsequently, the crossover is performed on the variables in the corresponding dimensions to generate new offspring. Assuming the *m*-th and *n*-th individuals are paired, the crossover operation is performed as follows:

(18) $$Mhc_{m,j}=r_{1}\cdot X_{m,j}+(1-r_{1})\cdot X_{n,j}+c_{1}\cdot (X_{m,j}-X_{n,j}),$$

(19) $$Mhc_{n,j}=r_{2}\cdot X_{n,j}+(1-r_{2})\cdot X_{m,j}+c_{2}\cdot (X_{n,j}-X_{m,j}),$$

where *Mhc_m,j_* and *Mhc_n,j_* are descendants of *X_m,j_* and *X_n,j_*, respectively. And *X_m,j_* and *X_n,j_* are two random individuals in the population. *r*_1_ and *r*_2_ are randomly distributed evenly between 0 and 1. *c_1_* and *c_2_* are randomly distributed evenly between −1 and 1.

The first term in Equations (18) and (19) represents the particle’s current optimum, and the second term represents the mutual influence between two different particles, and these two terms are well combined through the weight factor *r*_1_. The third term can increase the search interval. The final solutions *Mhc_m,j_* and *Mhc_n,j_* must be compared with the fitness of the parent particles *X_m,j_* and *X_n,j_*, and the solution with better fitness should be retained for the next iteration.

#### 3.4.2. Vertical Crossover

Vertical crossover is executed across distinct dimensions of the variable. Due to the different ranges of values for different dimensions, they need to be normalized before crossing. Each vertical crossover only generates one offspring, and only updates one dimension of it.

(20) $$Mvc_{m,d_{1}}=r\cdot X_{m,d_{1}}+(1-r)\cdot X_{m,d_{2}},$$

where *r* is randomly distributed evenly between 0 and 1.

Vertical crossing can cause the dimension that has already fallen into a local optimum to escape from local optimality without damaging the information of the other dimension. Thus, in general, this strategy is effective in keeping population sizes from dropping into local minima, and the probability of a vertical crossover is lower than the probability of a horizontal crossover.

#### 3.4.3. Competitive Operator

There is a competitive relationship between the offspring population and the parent population. Only if the adaptation value of the offspring population is preferred to that of the parent population will it be retained and proceed to the next iteration. Otherwise, the parent population will continue to be retained. As a result of this simple competitive mechanism, individuals will move rapidly toward the search space with good fitness, close to the optimal solution. For example, in terms of horizontal crossing, the competition operator is defined as

(21) $$X_{m}^{offspring}=\left\{\begin{array}{l} X_{m},\;\;f(X_{m})<f(Mhc_{m})\\ Mhc_{m},\;\mathrm{else} \end{array}\right..$$

### 3.5. The Framework of CMRLCCOA

Inspired by the above strategies (chaotic mapping, lens imaging reverse learning, Lévy flight, and crossover strategy), we propose a new hybrid metaheuristic algorithm, CMRLCCOA. These strategies greatly strengthen the stability and optimization capability of the algorithm. The specific steps for solving the D-dimensional minimum problem using CMRLCCOA are as follows:

Step 1: Initialize some parameters of CMRLCCOA—the number of search agents *N*, dimension of the solution *D*, boundaries of variables *ub* and *lb*, and number of iterations *M_iter_*.

Step 2: Initializing *N* populations of coatis using chaotic mapping.

Step 3: The fitness values for each candidate solution are computed. Afterwards, record the best fitness value *f_best_* and the optimal position *X_best_*.

Step 4: Using a convex lens imaging reverse learning strategy to update *N* initial solutions by Equation (11), then calculating fitness values while retaining good fitness values and optimal solutions.

Step 5: While *C_iter_* < *M_iter_*, update the location of the iguana.

Step 6: For the first half of the individual coatis, using Equation (2) to change location of the *i*-th coati, and using Equation (7) again to update the position of the *i*-th coati.

Step 7: For the latter half of the individual coati, first set the iguana’s random location using Equation (3), then use Equation (4) to compute the new position of the *i*-th coati, and finally use Equation (7) to update the position of the *i*-th coati.

Step 8: Utilizing the Lévy flight strategy, the coati’s position is updated by Equation (18) and candidate solutions are calculated, while retaining the optimal solution and corresponding position.

Step 9: In the second stage of exploitation, first calculate the local boundaries of variables by Equation (5). The location of the *i*-th coati is changed using Equation (6). Equation (7) is used to update the optimal solution.

Step 10: Using the cross optimization strategy, horizontal and vertical crosses are performed on individuals of the coati by Equations (19)–(21) and offspring populations are obtained, and then the better preserved ones are selected from the parent and offspring populations.

Step 11: Set *C_iter_* = *C_iter_* + 1; if *C_iter_* < *M_iter_*, return to Step 5. Otherwise, the optimal location and fitness values obtained from solving the problem will be output.

**Figure 8:**
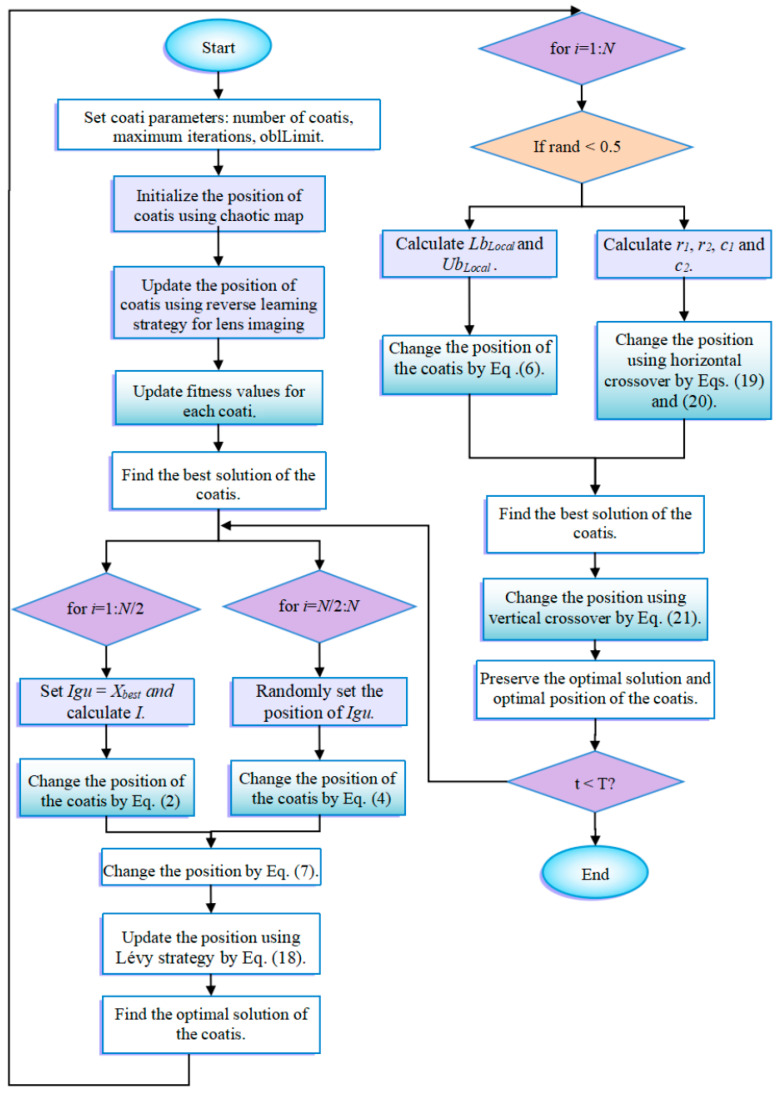
Flowchart of CMRLCCOA.

To show the structure of the CMRLCCOA more clearly, the flowchart of CMRLCCOA is illustrated in Figure 8. Additionally, the pseudo-code of CMRLCCOA is shown in Algorithm 1.
**Algorithm 1:** The proposed CMRLCCOA**Input:** Number of coatis (*N*), Number of variables (*D*), and maximum iterations (*M_iter_*).**Output:** Optimal fitness value *f_best_ and X_best_.*1: Construct the initial value for the agents through chaotic maps.2: Computing fitness values for coati populations.3: Using convex lens imaging reverse learning strategy to change the coatis’ position by Equation (11).4: Compare fitness values and retain the optimal fitness values and corresponding positions.5: **While** *t* ≤ *M_iter_*6:    **For** *i* = 1 to *N*/27:      *Igu* = *X_best_*; *I = round*(1 *+ rand*(1,1)).8:      Change the position of the coati by *x_i,j_ = x_i,j_ + b* × (*Igu_j_* − *I*×*x_i,j_*).9:      Update position by Equation (7).10:   **End For**11:   **For** *i* = *N*/2 to *N*12:     *Igu* = *lb* + *rand* × (*ub* − *lb*).13:      **If** fitness(*i*) > fitness(*Igu*)14:        Change the position by *x_i,j_* = *x_i,j_* + *b* × (*Igu_j_* – *I* × *x_i,j_*).15:      **Else**
16:        Change the position by *x_i,j_* = *x_i,j_* + *b* × (*I* × *x_i,j_* − *Igu_j_*).17:      **End If**18:     Update position by Equation (7).19:   **End For**20:   Using Lévy strategy to update the position of the *i*-th coati by Equation (18).21:   Calculate the fitness of coatis.22:   **If** the fitness of coati < fitness(*i*)23:      *x*(*i*) = coati;fit(*i*) = fit(*coati*).24:   **End If**
25:   **For** *i* = 1 to *N*26:      *Lb_Local_* = *lb/t*; *Ub_Local_* = *ub/t.*27:        **If**
*rand* < 0.528:        Update the position of the coatis by *x_i,j_* = *x_i,j_* + (1 − 2*b*) × (*Lb_Local_* + *b* × (*Ub_Local_* − *Lb_Local_*)).29:        Update position by Equation (7).30:      **Else**
31:         **For**
*j* = 1 to *D*32:            *r*_1_ and *r*_2_ is a stochastic number in [0, 1]; *c*_1_ and *c*_2_ is a stochastic number in [−1, 1].33:            Update the position of the leaders using Equations (18) and (19).34:            Calculating acclimatization values of coatis.35:         **End For**36:      **End If**37:   **End For**38:   **For**
*i* = 1 to *N*-139:      **For**
*j* = 1 to *D*40:         Update a uniformly random value *r* in 0 and 1.42:         Update the position of the individuals using Equation (20).43:         Calculating acclimatization values of coatis.44:      **End For**45:   **End For**46:   *t* = *t* + 1,47: **End While**

### 3.6. The Time Complexity of CMRLCCOA

This subsection investigates the time complexity of CMRLCCOA. First, we analyze the COA. The population scale and the amount of problem variables mainly contribute to the time complexity. In the initialization phase, the complexity of COA is O(ND), where *N* is the size of the coati population and *D* is the amount of variables. Among four improvement strategies for the presented CMRLCCOA, the chaotic mapping and lens imaging reverse learning strategies do not increase the complexity. In the first stage, the time complexity is O(NDT) + O(NDT/2) + O(NDT). In the second stage, the complexity is O(NDT) + O((N − 1)DT). Thus, the complexity can be characterized as Equation (22).

*O*(CMRLCCOA) = *O*(Origin coatis) + *O*(Hunting) + *O*(Escaping) = *O*(*ND*(1 + 5/2*T*) + (*N* − 1)*DT*) (22)

## 4. Numerical Experiments and Comparison with Other Algorithms

In Section 4, we conduct experiments using functions from the CEC2017 and CEC2019 test suites. Among them, it contains 29 functions for CEC2017 and 10 functions for CEC2019. The number of iterations in this experiment is 500 and thirty individuals constitute the entire population. The dimensions of CEC2017 are 50 and 100. The CMRLCCOA is compared to fourteen existing metaheuristic algorithms, including four recognized classical algorithms, PSO (particle swarm optimization) [15], DE (differential evolution) [12], SA (simulated annealing) [16], and ABC (Artificial Bee Colony Algorithm) [61]; six recently proposed algorithms, KOA (Kepler Optimization Algorithm) [62], SWO (Spider Wasp Optimizer) [63], GMO (Geometric Mean Optimizer) [64], OMA (Optical Microscope Algorithm) [65], TROA (Tyrannosaurus Optimization Algorithm) [66], and GO (GOOSE Algorithm) [67]; and three improved algorithms, ISSA (Improved Sparrow Search Algorithm) [68], IGWO (Improved Grey Wolf Optimizer) [69], and EWOA (Enhanced Whale Optimization Algorithm) [70]. Each comparison algorithm is run independently for 20. Finally, the optimum value, the worst value, the mean value, the standard deviation, and the rank for all results are calculated. Furthermore, the Wilcoxon signed rank test is performed to further check the quality of CMRLCCOA. The parameters of the other metaheuristic algorithms are listed in Table 2. Finally, all tests are experimented in Matlab-2020b with a 2.11 GHz quad-core Intel(R) Core(TM) i5 and 8.00 GB.

### 4.1. Introduction to Test Sets

A total of 39 functions were used for testing in this experiment, which come from CEC2017 [71] and CEC2019 [72].

**Figure 9:**
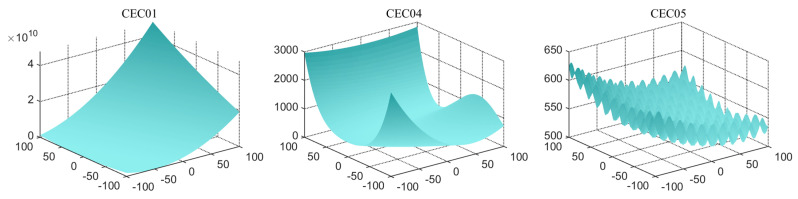

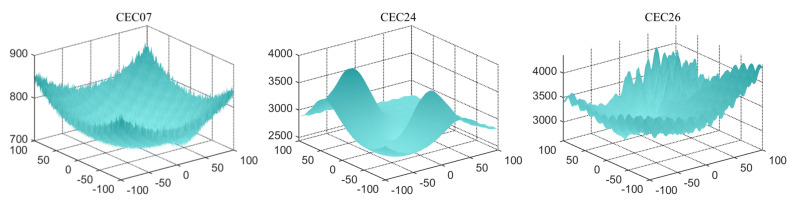
The partial function diagrams in CEC 2017.

CEC2017 is a test set of intelligent algorithms widely used for a number of optimization problems. The functions are rotated and translated, which in turn increases the difficulty of finding optimization for the algorithms and has a high degree of acceptance. There are four types of benchmark functions: single-peaked, multi-peaked, hybrid, and combined. The single-peak functions (cec01, cec03) are characterized by the fact that there is only a global minimum, not a local minimum. This type of function verifies the convergence of the algorithm. Multi-peak functions (cec04–cec10) have local minima. Such functions verify the competence to get rid of local optima. Algorithms that perform well on these functions generally possess strong exploration capabilities. Hybrid functions (cec11–cec20) have each sub-function assigned a certain weight, which in turn better combines the properties of each sub-function. These functions can effectively verify the ability to find the global optimum. Composite functions (cec21–cec30) have additional bias values and weights for each sub-function. Such functions allow us to assess the accuracy of algorithms. The comprehensive performance will be demonstrated on these functions. To show the details of these functions more clearly, the partial function diagrams are shown in Figure 9.

**Figure 10:**
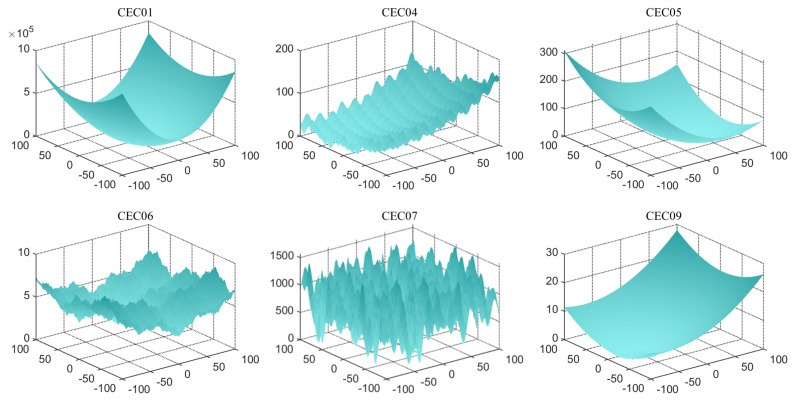
The partial function diagrams in CEC 2019.

In addition, this experiment also uses the CEC2019 test set to assess the algorithm’s capability. The CEC2019 test set [73] is a very effective benchmark function set for metaheuristic algorithm performance testing. Among them, cec01–cec03 have different dimensions and ranges, and cannot be moved and rotated. cec04–cec10 are ten-dimensional minimization problems, which can be moved and rotated. This test set is known as the “100-bit challenge” and is often used in international competitions. Some of the functions of CEC 2019 are shown in Figure 10.

### 4.2. Assessment of Indicators

To appraise the efficacy of all algorithms, we take several performance metrics for the analysis, which include the optimal value (Best), the worst value (Worst), the mean value (Ave), the standard deviation (Std), and Rank. Their equations are presented in Equation (23) to Equation (26). The comparison of these metrics can be used to analyze the performance. It is worth noting that Ave can indicate the precision of the algorithm when addressing specific problem categories. The stability can be obtained from Std. Rank is obtained by comparing Ave and Std. If Rank is smaller, it means that the algorithm has a superior performance in solving a particular problem.

(1) Optimum value (Best)

(23) $$\mathrm{Best}=\underset{1\leq i\leq m}{\min}f_{i}^{*}$$

(2) Worst value (Worst)

(24) $$\mathrm{Worst}=\underset{1\leq i\leq m}{\max}f_{i}^{*}$$

(3) Mean value (Ave)

(25) $$\mathrm{Ave}=\frac{1}{m}\sum_{i=1}^{m}f_{i}^{*}$$

(4) Standard deviation (Std)

(26) $$\mathrm{Std}=\sqrt{\frac{1}{m-1}\sum_{i=1}^{m}{\left(f_{i}^{*}-\mathrm{Ave}\right)}^{2}}$$

where *m* corresponds to the tally of independent runs of the algorithms. f_i_* is the global optimum obtained at the *i*-th independent run.

### 4.3. Effect of Different Chaotic Mapping Functions on CMRLCCOA

**Figure 11:**
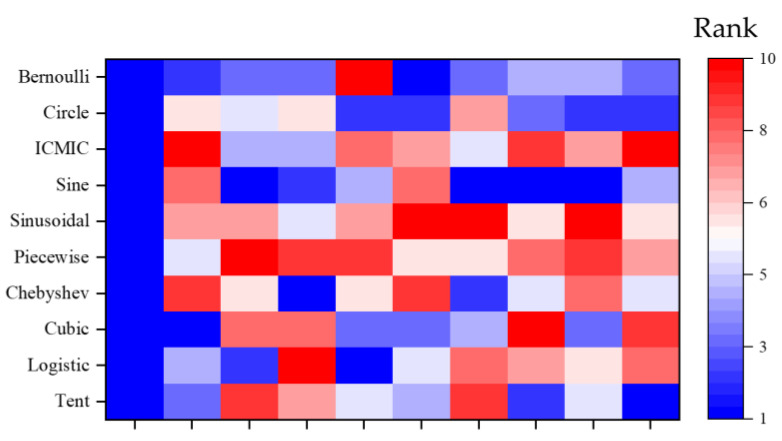
Heat maps corresponding to the rankings obtained from different chaotic mappings.

Chaotic mapping produces a random sequence for initializing the population, producing a better initial solution [74]. Chaotic mapping produces initial populations in different ways and gives different results. A better strategy produces good initial solutions and is a great enhancement for the subsequent optimization of the algorithm. In this context, 10 functions (taken from CEC2019) are optimized using 10 of the more common chaotic mapping methods to analyze and compare the impact of different chaotic mappings. Table 1 lists these 10 recognized chaotic mapping methods. Table 3 displays the effect and ranking of the 10 chaotic mapping strategies on CMRLCCOA. From the data, it can be seen that the Sine mapping strategy has the best optimization result with the smallest total rank, followed by the Bernoulli mapping strategy and Circle mapping strategy. From the above analysis, it can be concluded that the Sine chaotic mapping strategy performs best in this method and is the first strategy in this method. To show the comparison results more clearly, the experimental data are visualized here, as shown in Figure 11, where the horizontal coordinate is the function of the test set and the vertical coordinate is the algorithm obtained through different mapping methods. And Figure 11 indicates that the Sine chaotic mapping strategy ranks first among the four functions. This also shows that these strategies can be relatively more effective in improving the performance of COA.

### 4.4. Comparison of Optimization Results for CEC2017 

For the purpose of examining CMRLCCOA’s competence in exploring, developing, and jumping out of local optimal solutions, a more competitive test suite, the CEC2017 test suite, was chosen for this paper. CEC2017 [71] is highly recognized and is widely used to validate the performance in all aspects. What is more, this function is not included in this paper because it was not possible to test cec02.

#### 4.4.1. Experimental Results of the CEC2017 Test Suite

In this experiment, CMRLCCOA is run 20 times independently with 14 other algorithms, and finally, Ave, Std, and Rank are calculated. Table A1 and Table A2 show the results obtained by 15 algorithms optimized in 50 and 100 dimensions. The top-ranked values are highlighted in a thick format. 

The observation of the tabular data shows that the CMRLCCOA is ranked first overall with an average ranking of 3 and 2.6552 for *dim* = 50 and 100, respectively; this result shows that the improved CMRLCCOA is better at optimizing in different dimensions and all of them provide excellent output values. Most of the optimal values obtained by CMRLCCOA computation outperform other algorithms. This phenomenon shows that CMRLCCOA is adaptable to different types of functions. CMRLCCOA is able to optimize the nine test functions better in *dim* = 50 (cec03, cec11–12, cec21, cec23, and cec27-30). At *dim* = 100, CMRLCCOA better optimizes 13 test functions (cec03, cec04, cec07, cec11–12, cec14, cec15, cec20–21, cec23–24, cec27–29). GMO also optimizes better and performs better for 10 50-dimensional problems and 100-dimensional problems, second only to CMRLCCOA, and ranked second overall. On the contrary, COA, KOA, SWO, TROA, and GO do not show better optimization ability. In summary, CMRLCCOA significantly outperforms COA as well as the other 13 intelligent optimization algorithms in 50 and 100 dimensions.

The Wilcoxon signed rank test verifies the variability of results obtained by different algorithms [75]. The significance results for *dim* = 50 and 100 are shown in Table A3 and Table A4. “+/=/-” means that the comparative algorithm is significantly better/equal/worse than the CMRLCCOA. The observation of the data reveals that the Wilcoxon test results for COA, KOA, SWO, TROA, and GO are 0/0/29 at *dim* = 50 and 100, indicating that these five algorithms are inferior to CMRLCCOA in all test functions. Meanwhile, the Wilcoxon signed rank results of GMO in two dimensions are 6/10/13 and 6/7/16, which are better. Secondly, ISSA and IGWO also perform better; both of them have six functions better than CMRLCCOA. However, when *dim* = 50 or 100, ABC, EWOA, SA, and DE algorithms perform significantly worse than CMRLCCOA. Therefore, this result also shows that CMRLCCOA can address different types of problems.

**Figure 12:**
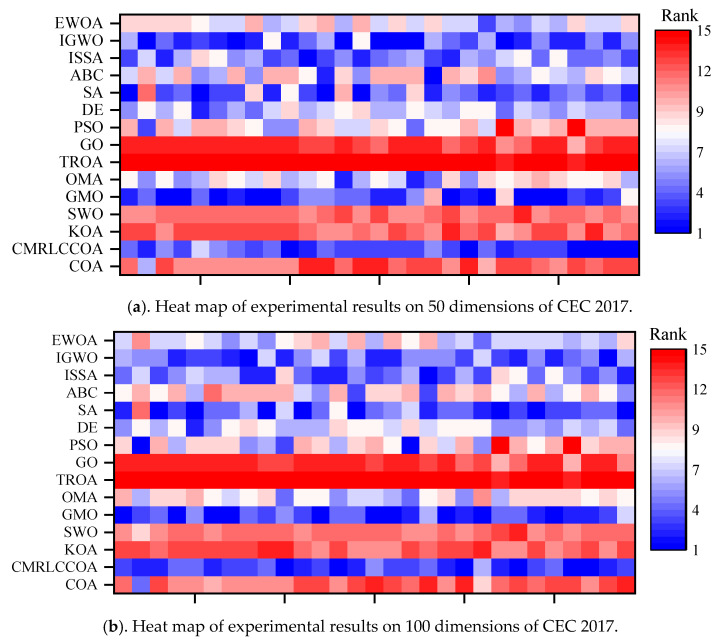
Heat map of experimental results on CEC 2017.

Similar to Figure 11, Figure 12 shows two heat maps of the results obtained for all compared algorithms on CEC 2017 for two different dimensions. The vertical coordinates are all algorithms involved in comparison. The performance of all algorithms can be intuitively obtained from the heat map. In the heat map of both dimensions, the corresponding squares of CMRLCCOA proposed in this paper largely show a bluer situation relative to other algorithms. Meanwhile, the GO and TROA rows are always red. This phenomenon indicates that these two algorithms perform poorly on this test set and their performance needs to be improved.

#### 4.4.2. Convergence Curves for Iterations

**Figure 13:**
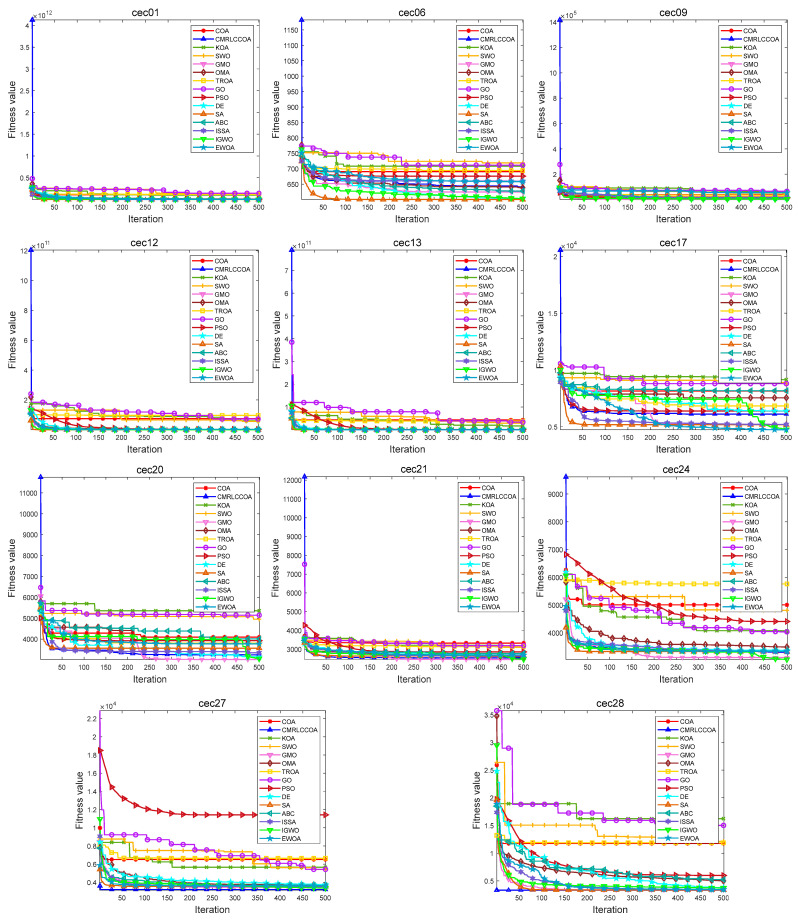
The iteration profile of CMRLCCOA with other comparative algorithms for *dim* = 50.

**Figure 14:**
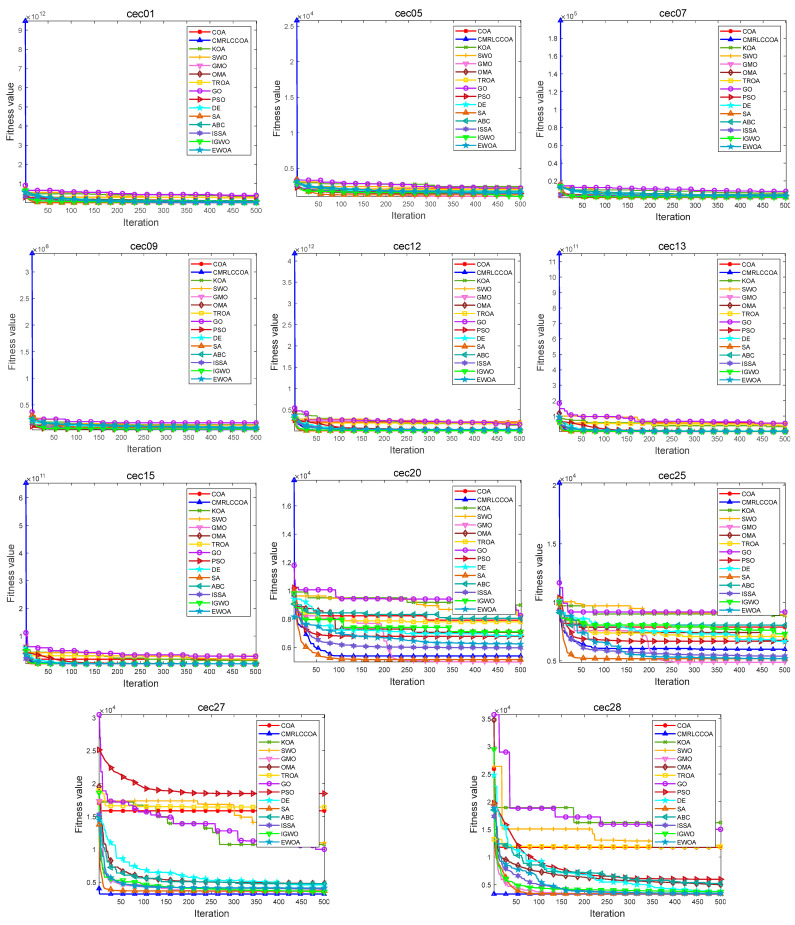
The iteration profile of CMRLCCOA with other comparative algorithms for *dim* = 100.

Figure 13 and Figure 14 show partial convergence curves at *dim* = 50 and 100. From the figure, it can be concluded that CMRLCCOA converges better on cec09, cec20–21, cec24, cec27, and cec28 at *dim* = 50. When *dim* = 100, CMRLCCOA converges better on functions cec07, cec09, cec12, cec20, cec25, and cec27–28. In addition, it can be seen that CMRLCCOA has a larger slope of the curve during the iteration of most functions, which indicates that CMRLCCOA always can converge faster in the early stages. This is made possible by the inclusion of the initialization strategy, which allows the population to explore a larger area. It is able to converge to the neighborhood of the optimum very quickly. In summary, CMRLCCOA can find the optimal solution quickly and can solve some sophisticated optimization questions.

The optimization ability of CMRLCCOA varies when dealing with different functions. As can be noticed from Figure 13 and Figure 14, CMRLCCOA is able to avoid interference factors well in the optimization of both single-peak and multi-peak functions, and both of them converge rapidly to the vicinity of the optimal solution. CMRLCCOA converges faster in hybrid and composite functions. The observation of the curves reveals that the slope of the pre-curve is very large and almost vertical so that the optimal candidate solution can be found in fewer iterations, which indicates that the algorithm has high sensitivity. In addition, it can be found that CMRLCCOA maintains the stability and continuity during the iteration process of most functions, and the convergence accuracy is better. In short, CMRLCCOA is able to solve the functions in CEC2017 efficiently.

#### 4.4.3. Boxplot of Experimental Results

**Figure 15:**
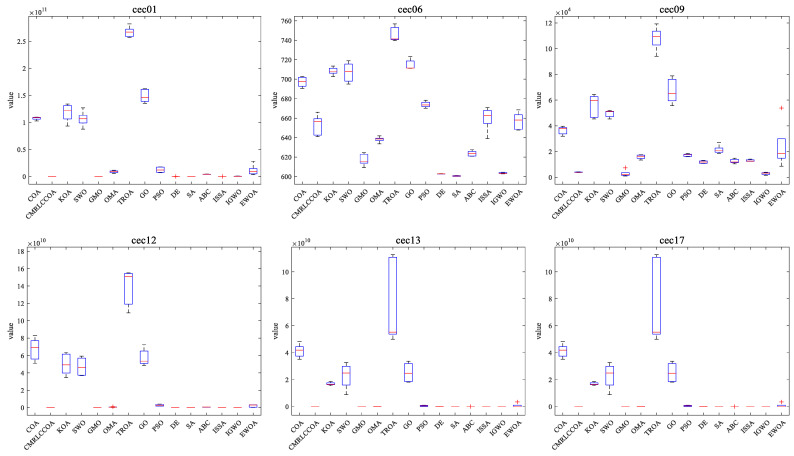

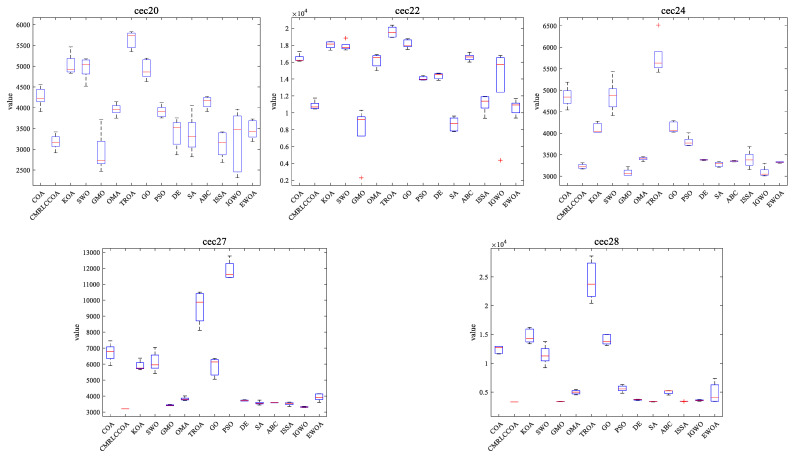
Boxplot of CMRLCCOA algorithm with other comparative algorithms for *dim* = 50.

**Figure 16:**
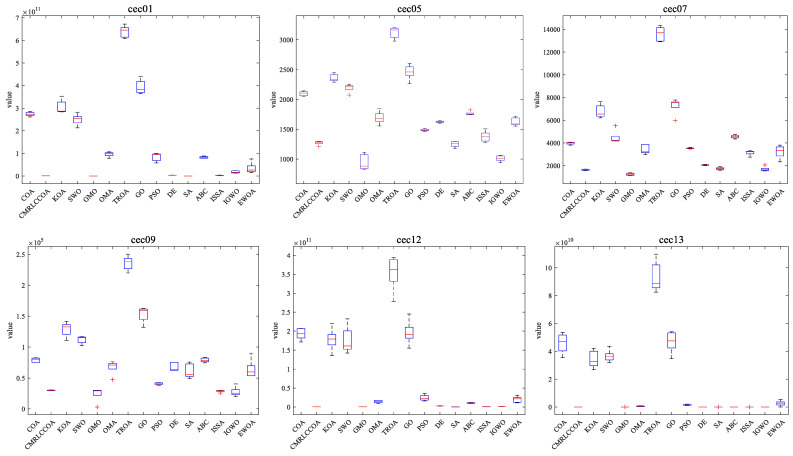

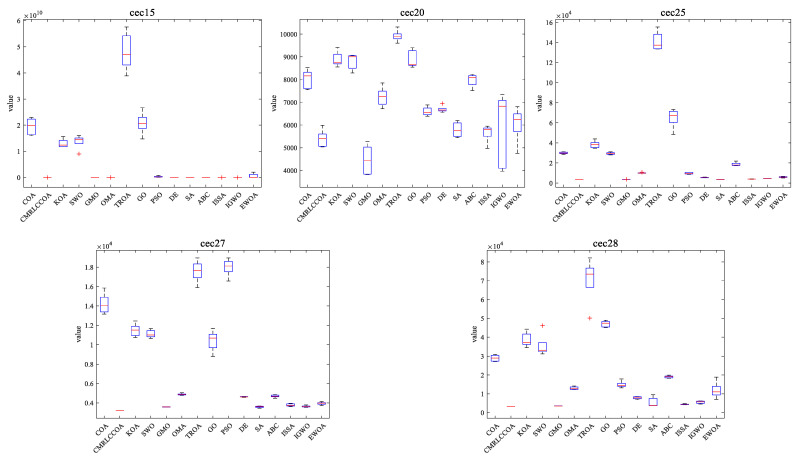
Boxplot of CMRLCCOA algorithm with other comparative algorithms for *dim* = 100.

Combined with the convergence curves above, the corresponding box plots are given here. A box-and-line plot is an icon that describes the discrete distribution of data and provides a good description of outliers and skewness in the data. The length of the boxes corresponds to the stability of algorithms. If the box is narrower, the algorithm is more stable and robust. The upper limit of the box-and-line plot is the upper quartile, and the lower line is the lower quartile. Because of the randomized nature of the algorithm, some outliers are generated during the optimization of the problem, and to visually demonstrate the quality of the optimization results, box-and-line plots of the optimization results at *dim* = 50 and 100 are given, as shown in Figure 15 and Figure 16. CMRLCCOA has less variation in the upper and lower distances than the other algorithms, especially at *dim* = 50 for cec22, cec24, cec27, and cec28, and at *dim* = 100 for cec01, cec09, cec11, cec12, ce25, and ce27–28. These functions verify the stability of CMRLCCOA. However, CMRLCCOA also shows some “+” indicators in the box plots of some functions, indicating that the algorithm also produces some outliers with uncertainty and randomness.

In conclusion, for most of the functions, CMRLCCOA is shorter and the upper and lower boundaries are closer together compared to the other 14 algorithms, which indicates that CMRLCCOA is more stable and has better minimum values compared to others.

### 4.5. Comparison of Optimization Results for CEC2019 

This part of the numerical experiments is performed using the 10 functions in CEC2019 [76]. First, the experiment was set up to run 20 independent repetitions. After that, the Ave, Best, Worst, Std, and Rank of the 20 results are computed. Secondly, we obtain the convergence iteration diagrams during the algorithm runs. In addition, all comparison algorithms are consistent with the above experiments. Table 4 illustrates the calculation results. First-ranked data are marked in bold. Finally, box-and-line plots are plotted as a visualization of the quality of the solution results. The radar plot shows more visually how each algorithm ranks on each test function.

#### 4.5.1. Statistical results on CEC2019 

As indicated in Table 4, the average rank of CMRLCCOA is 2.3, which is ranked first overall, better than the others and significantly better than COA. This effect indicates that CMRLCCOA obtains solutions of higher quality relative to the others. In addition, CMRLCCOA significantly optimizes the five functions (cec01, cec04, cec05, cec07, cec08). CMRLCCOA ranks first in terms of computational results in cec01, indicating that it performs well in low-dimensional test functions. CMRLCCOA is superior to the others in cec04 and cec05. This indicates that it is also suitable for higher-dimensional test functions. CMRLCCOA has excellent optimization ability in cec07 and cec08. GMO performs excellently in completing some problems, and has excellent optimization ability, ranking second. GMO outperforms the other algorithms and has a strong optimization ability. In contrast, other algorithms do not solve these functions well.

Table 5 shows the final test results for the Wilcoxon signed rank [77]. A look at the data in Table 5 reveals that the Wilcoxon symbolic rank test outputs for COA, KOA, SWO, GMO, OMA, TROA, GO, PSO, DE, SA, ABC, ISSA, IGWO, and EWOA are 0/1/9, 0/0/10, 0/0/10, 3/2/5, 1/1/8, 0/0/10, 0/0/10, 2/2/6, 2/3/5, 0/3/7, 0/1/9, 2/2/6, 2/1/7, and 0/1/9. It can be found that KOA, SWO, TROA, and GO are not as good as CMRLCCOA on all the tested functions, which can show that CMRLCCOA has better performance and is competitive.

#### 4.5.2. Convergence Curves for Iterations

**Figure 17:**
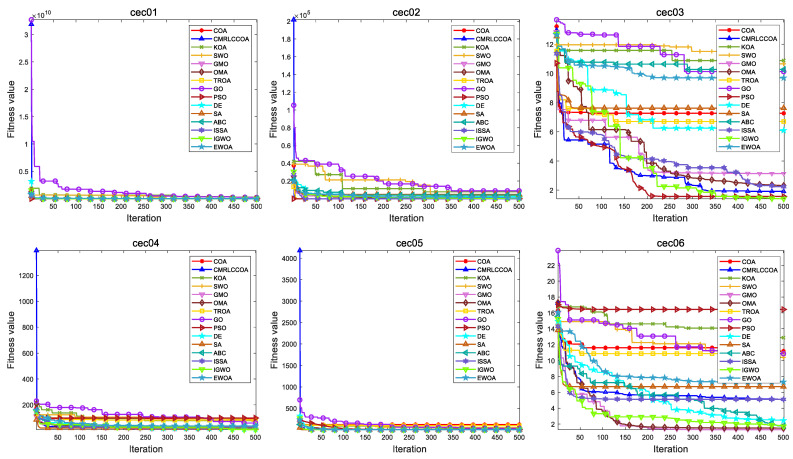

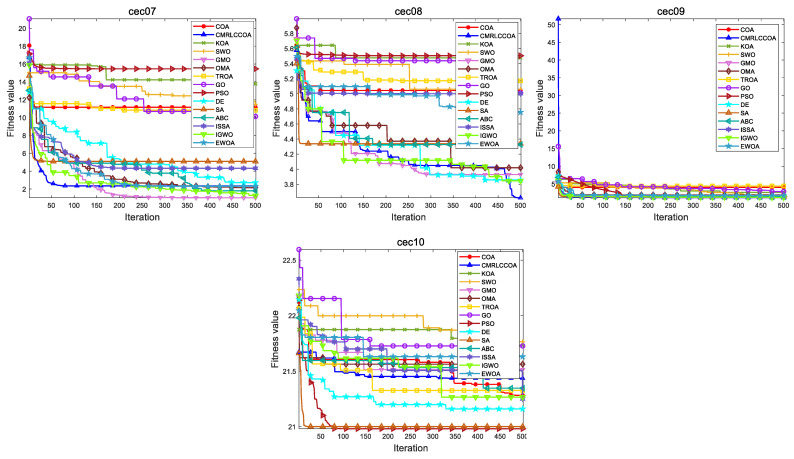
The iteration profile of CMRLCCOA with other comparative algorithms in CEC2019.

The convergence curves of comparison algorithms are shown in Figure 17. CMRLCCOA has a very smooth iteration curve and can approach the optimal solution quickly. This shows that CMRLCCOA converges faster than other algorithms, so this algorithm is capable to solve high- and low-dimensional problems. In the experiments on the high-dimensional cec03 function, the CMRLCCOA function converges very fast and moves rapidly to the optimal solution. The CMRLCCOA reaches the neighborhood of the optimum with few iterations during the solution of functions cec05, cec07, and cec09. The convergence is significantly better. The GMO for cec08 is closest to the optimal value and its convergence effect is also excellent. In addition, as shown in Figure 12, the CMRLCCOA algorithm has a very large slope, almost vertical, on the early convergence curves of most functions, indicating that the algorithm has a high sensitivity. Also, PSO, IGWO, OMA, and GMO algorithms show good competence in certain functions. The results show that CMRLCCOA converges faster, gradually approaches the optimal solution, and has better optimization ability than others.

#### 4.5.3. Boxplot of Experimental Results

**Figure 18:**
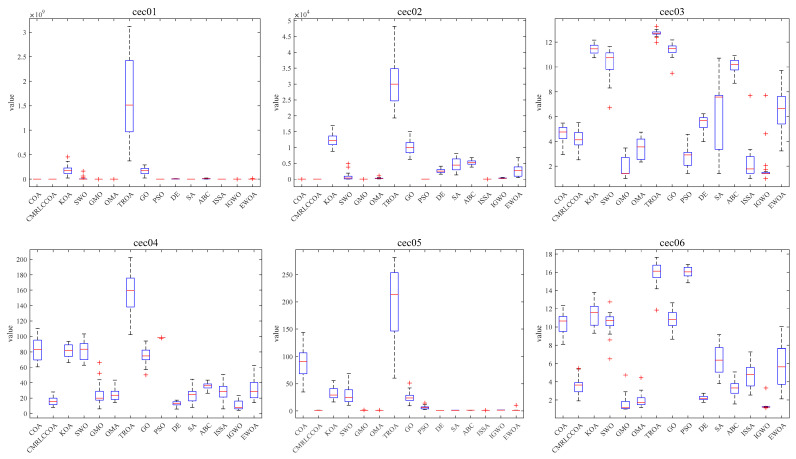

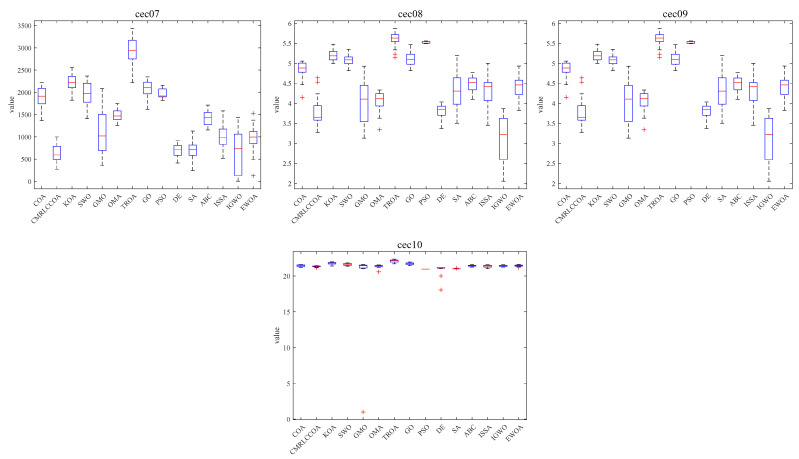
Boxplot of CMRLCCOA algorithm with other comparative algorithms in CEC2019.

Figure 18 illustrates a box-and-line plot of CMRLCCOA and other comparative algorithms optimizing the CEC2019 test function. As can be noticed from the figure, it can be noticed that the CMRLCCOA has a lower median case and narrower inter-quartile range, especially in the functions cec01, cec02, cec05, and cec10. It shows that the solutions of CMRLCCOA are more centralized than the other algorithms and are robust. However, CMRLCCOA produces outliers in the optimization process of some functions, such as cec08 and cec09. This phenomenon indicates that this algorithm is unstable to some extent.

#### 4.5.4. Radargram Behavior Analysis

**Figure 19:**
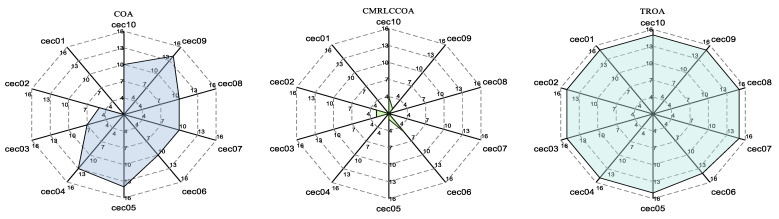

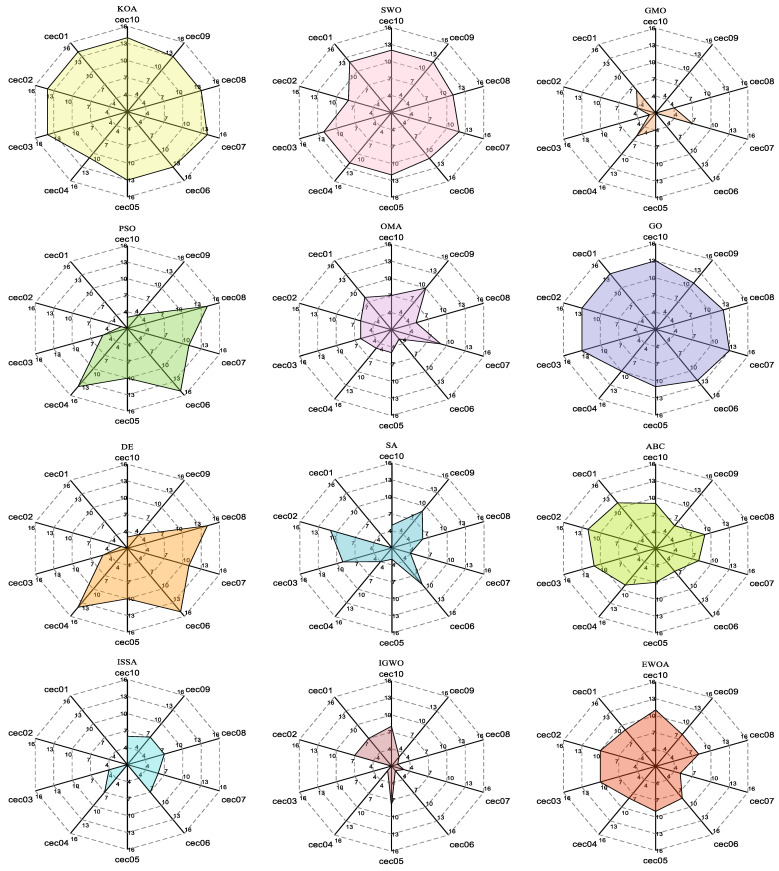
Radar chart of CMRLCCOA and other algorithms on CEC2019.

A radar chart is a chart that shows multidimensional data and also shows how much weight is given to each variable in a set of data and can be used to show performance data. To visualize the performance ranking of the different tested functions for all algorithms, Figure 19 illustrates the radar chart of the results for the 10 tested functions sorted. A larger area of the filled portion indicates a lower overall ranking of the algorithm. From Figure 19, it can be concluded that CMRLCCOA has the smallest area, which indicates that CMRLCCOA has the smallest total ranking and the best overall optimization capability. Furthermore, GMO and IGWO also show better performance.

**Figure 20:**
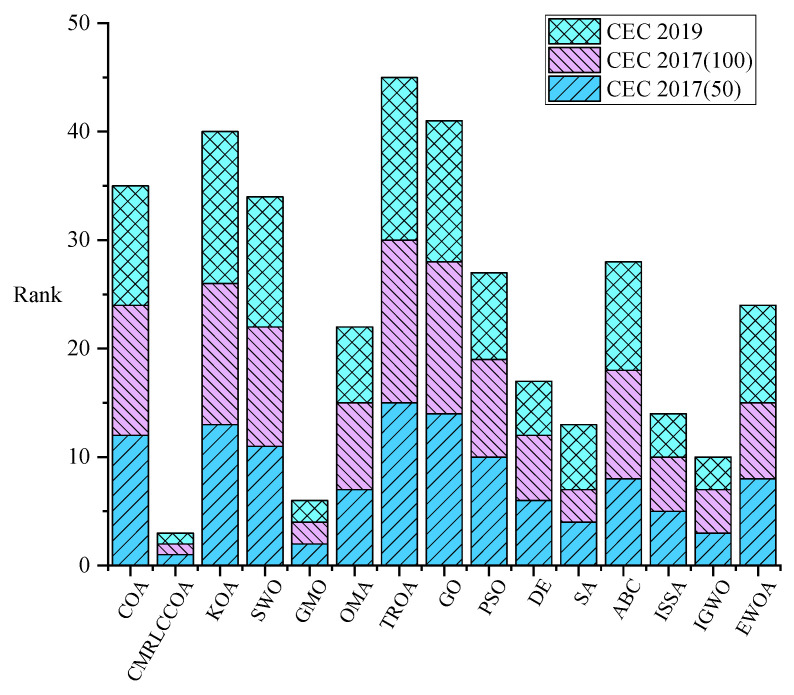
The stacked histogram on all test sets.

To show the results on the test set more clearly, they are shown here by stacked histograms. As shown in Figure 20, the total height of CMRLCCOA is the lowest. This indicates that CMRLCCOA has relatively the best overall performance and CMRLCCOA is effective. This shows that the mixing of multiple strategies with COA and the construction of CMRLCCOA are effective as well as successful.

## 5. Solutions to Real-World Engineering Optimization Problems

To further test the performance of CMRLCCOA, this section tests it against several excellent metaheuristic algorithms on several complex real-world engineering applications. The comparison algorithms include KOA [62], TROA [66], BWO [29], AO [78], HBA [79], SWO [63], GMO [64], OMA [65], and GO [67]. Their parameters are kept consistent with Table 2. They are not repeated here. The experimental part is as follows.

### 5.1. Single-Stage Cylindrical Gear Reducer (SSCGR)

**Figure 21:**
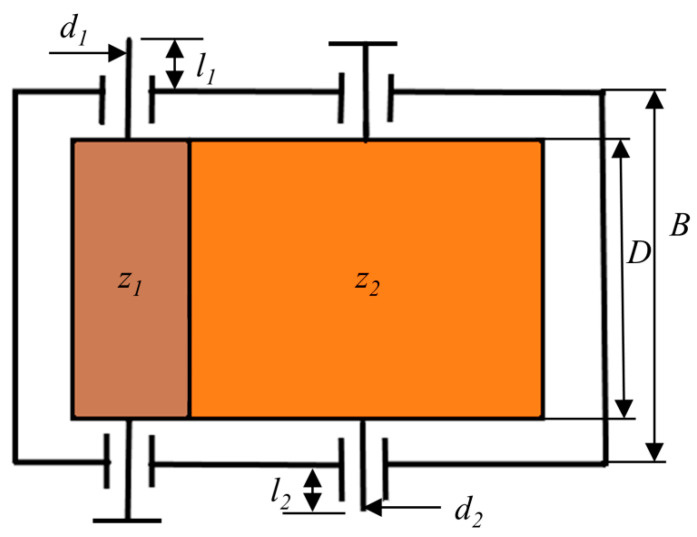
Plane schematic diagram of SSCGR.

SSCGR [80] is a kind of reducer that is widely used. The reducer consists of an input shaft and an output shaft. It typically serves as a speed reducer between the primary component and the operational machinery. However, designers tend to focus on the quality and design efficiency of the reducer, thus ignoring the reducer’s consumables, resulting in a huge waste. With the goal of minimizing the number of reducer consumables, SSCGR is established as follows under the premise of meeting the physical model and stability requirements of the reducer: tooth width D, case width B, discrete parameter modulus P and three integer parameters, $\left\{d_{1},d_{2},z_{1}\right\}$, namely. Figure 21 illustrates a diagram of the SSCGR. After specifying the optimization objective, we define the problem as follows:

$$X=\left[D,B,P,d_{1}^{\prime },d_{2}^{\prime },z_{1}\right].$$

Minimize

$$\begin{array}{ll} f(X) & =\pi (1.1875x_{1}x_{3}^{2}x_{6}^{2}+0.262x_{1}x_{4}^{2}-0.282x_{1}x_{5}^{2})\\ \; & +21.25x_{1}x_{3}^{2}x_{6}+0.25x_{2}x_{4}^{2}+0.25x_{2}x_{5}^{2}+70x_{4}^{2}\\ \; & +80x_{5}^{2}-21.25x_{1}x_{3}^{2}+0.2x_{1}x_{3}x_{5}x_{6}-0.4x_{1}x_{3}x_{5}) \end{array}$$

subject to

$$g_{1}(X)=\frac{1,367,657.1038}{x_{3}x_{6}\sqrt{x_{1}}}-855\leq 0,\;g_{2}(X)=\frac{6,952,400,000}{-0.0854x_{1}x_{3}^{2}x_{6}^{3}+6.666x_{1}x_{3}^{2}x_{6}^{2}+169x_{1}x_{3}^{2}x_{6}}-261\leq 0,\;g_{3}(X)=\frac{6,952,400,000}{-0.394x_{1}x_{3}^{2}x_{6}^{3}+17.695x_{1}x_{3}^{2}x_{6}^{2}+2824x_{1}x_{3}^{2}x_{6}}-213\leq 0,\;g_{4}(X)=\frac{12.077149x_{2}^{3}}{x_{3}x_{4}^{4}x_{6}}-0.003x_{2}\leq 0,\;g_{5}(X)=\frac{28,456,113.636}{x_{3}x_{4}^{2}x_{6}}\sqrt{1+0.307380\frac{x_{3}^{2}x_{6}^{2}}{x_{2}^{2}}}-55\leq 0,\;g_{6}(X)=\frac{28,456,113.636x_{2}}{x_{3}x_{5}^{3}x_{6}}\sqrt{1+7.684501\frac{x_{3}^{2}x_{6}^{2}}{x_{2}^{2}}}-55\leq 0,\;g_{7}(X)=17-x_{6}\leq 0,\;g_{8}(X)=2-x_{3}\leq 0,\;g_{9}(X)=x_{3}x_{6}-300\leq 0,\;g_{10}(X)=16-x_{1}/x_{3}\leq 0,\;g_{11}(X)=x_{1}/x_{3}-35\leq 0,\;g_{12}(X)=100-x_{4}\leq 0,\;g_{13}(X)=x_{4}-150\leq 0,\;g_{14}(X)=130-x_{5}\leq 0,\;g_{15}(X)-200\leq 0,\;g_{16}(X)=x_{1}+0.5x_{5}+40-x_{2}\leq 0,$$

where the ranges of six design variables being $50\leq x_{1}\leq 150$, $150\leq x_{2}\leq 350$, $0\leq x_{3}\leq 50$, $50\leq x_{4}\leq 150$, $50\leq x_{5}\leq 200$, and $15\leq x_{6}\leq 30$. In addition, *x*_3_ is a discrete variable, the range of the variable is shown in Table 6, and *x*_4_, *x*_5_, and *x*_6_ are integer variables.

Table 7 and Table 8 contain the values of the variables taken and the minimum amount of consumables obtained by CMRLCCOA and other algorithms. It proves that CMRLCCOA outperforms other algorithms on average and is relatively stable. Therefore, CMRLCCOA is preferred for solving this hybrid discrete problem.

### 5.2. Welded Beam Design Problem (WBD)

**Figure 22:**
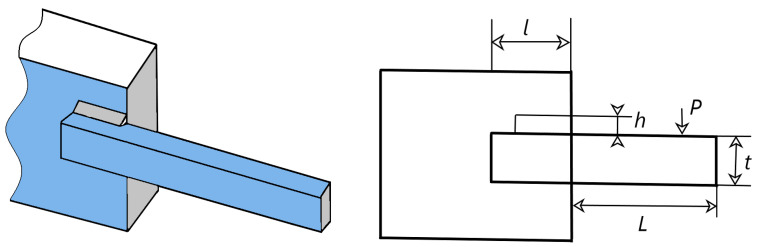
Schematic of WBD.

WBD [81,82] is a classical nonlinear programming problem. Its target is to reduce the production costs associated with the design. Figure 22 illustrates the WDB. This problem is to obtain four constraints that satisfy the constraints of shear stress (*τ*), bending stress (*θ*), bending load (*P*) of the beam bar, end deviation (*δ*), and boundary conditions, such that the cost of fabricating the welded beam is minimized. This question can be explained as follows:

Variant:

$$\vec{Q}=[q_{1},q_{2},q_{3},q_{4}]=[h,l,t,b].$$

Minimize

$$f(\vec{Q})=1.10471q_{1}^{2}q_{2}+0.04811q_{3}q_{4}(14.0+q_{2}).$$

subject to

$$f_{1}(\vec{Q})=\tau (\vec{Q})-\tau_{\max}\leq 0,\;f_{2}(\vec{Q})=\sigma (\vec{Y})-\sigma_{\max}\leq 0,\;f_{3}(\vec{Q})=\delta (\vec{Q})-\delta_{\max}\leq 0,\;f_{4}(\vec{Q})=q_{1}-q_{4}\leq 0,\;f_{5}(\vec{Q})=P-Pc(\vec{Q})\leq 0,\;f_{6}(\vec{Q})=0.125-q_{1}\leq 0,\;f_{7}(\vec{Q})=1.10471q_{1}^{2}+0.04811q_{3}q_{4}(14.0+q_{2})-5.0\leq 0.$$

Variable Scope:

$$0.1\leq q_{1}\leq 2,\;0.1\leq q_{2}\leq 10,\;0.1\leq q_{3}\leq 10,\;0.1\leq q_{4}\leq 2.$$

where

$$\tau (\vec{Q})=\sqrt{{\left(\tau^{\prime }\right)}^{2}+2\tau^{\prime }\tau^{\prime \prime }\frac{q_{2}}{R}+{\left(\tau^{\prime \prime }\right)}^{2}},\;\tau^{\prime }=\frac{P}{\sqrt{2}q_{1}q_{2}},\;\tau^{\prime \prime }=\frac{MR}{J},\;M=P(L+\frac{q_{2}}{2}),\;R=\sqrt{\frac{q_{2}^{2}}{4}+{\left(\frac{q_{1}+q_{3}}{2}\right)}^{2}},\;J=2\left\{\sqrt{2q_{1}q_{2}}\left[\frac{q_{2}^{2}}{4}+{\left(\frac{q_{1}+q_{3}}{2}\right)}^{2}\right]\right\},\;\sigma (\vec{Q})=\frac{6PL}{q_{4}q_{3}^{2}},\;\delta (\vec{Q})=\frac{6PL^{3}}{Eq_{3}^{2}q_{4}},\;P_{c}(\vec{Q})=\frac{4.013E\sqrt{\frac{q_{3}^{2}q_{4}^{6}}{36}}}{L^{2}}(1-\frac{q_{3}}{L}\sqrt{\frac{E}{64G}}),\;P=6000lb,\;L=14in,\;\delta_{\max}=0.25in,\;G=12\times 10^{6}psi,\;E=30\times 10^{6}psi,\;\tau_{\max}=13,600psi,\;\sigma_{\max}=30,000psi.$$

Table 9 presents the values of the variables and the manufacturing costs. Observing the graphs, it is noticeable that the mean and minimum expenses obtained by CMRLCCOA are less than the comparative algorithms. Therefore, CMRLCCOA can be prioritized when solving similar problems and this algorithm is significantly competitive.

### 5.3. Cantilever Beam Design Problem (CBDP)

**Figure 23:**
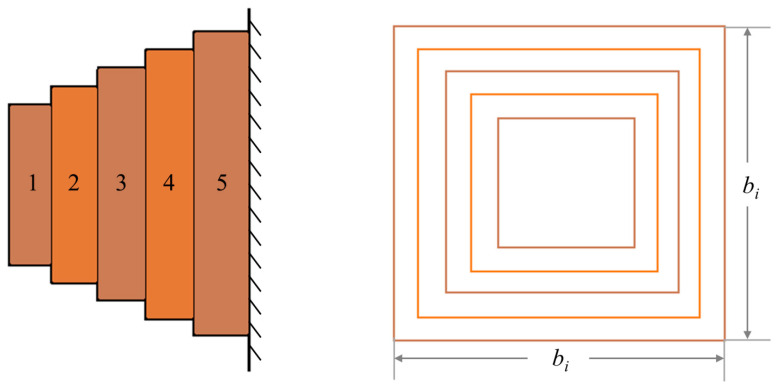
Illustration of CBDP.

This problem is to reduce the weight of the suspension beam arm. The structure of the cantilever beam consists of five hollow cells, each of which has the same cross-sectional thickness [83]. Figure 23 illustrates the structure. The thickness of the crossbar remains fixed and the variables are the widths of the five sections. The issue is presented as follows:

Minimize

$$f(b)=0.0624(b_{1}+b_{2}+b_{3}+b_{4}+b_{5}),\;b_{i}>0.$$

subject to

$$g_{1}(b)=\frac{61}{b_{1}^{3}}+\frac{37}{b_{2}^{3}}+\frac{19}{b_{3}^{3}}+\frac{7}{b_{4}^{3}}+\frac{1}{b_{5}^{3}}\leq 1.$$

Variable range:

$$0.01\leq b_{i}\leq 100,\;i=1,2,3,4,5.$$

Table 10 shows the values of the variables obtained from all algorithms, as well as the quality. The best results are marked in bold. Comparing the other results, CMRLCCOA obtains the minimum mass and the results are more stable. This phenomenon demonstrates that CMRLCCOA can solve the cantilever beam problem effectively.

## 6. Real Application: Engineering Optimization Problems

Hypersonic technology is an important milestone in the history of the world’s armaments and equipment, which greatly enriches the content of offensive and defensive confrontation in the adjacent space, represents a country’s ability to develop and utilize space in the future, and is an important symbol of the army’s combat power and survivability, and has a wide range of prospects for application and extremely important military value. The main advantages of hypersonic vehicles are fast flight speed, high flyable altitude from the ground, strong capability of surprise and defense, and great destructive power. In the face of future informationization and intelligent combat, hypersonic vehicles can play a great role by using their characteristics [84].

Since hypersonic vehicles are extremely fast in flight, the environment becomes more complex when the vehicle enters the re-entry or cruise phase, resulting in the need for a control system that is extremely stabilized and at the same time can achieve precise control. Due to the extremely high speed, the missile cannot make a sharp turn in the air. Therefore, in some instances, it is necessary to limit the curvature and turn rate of the aircraft trajectory [85]. Many scholars at home and abroad have also studied this problem. In this section, we will model the path planning of cruise missiles for hypersonic vehicles and apply CMRLCCOA to solve the problem [86].

### 6.1. Background and Establishment of the Model

**Figure 24:**
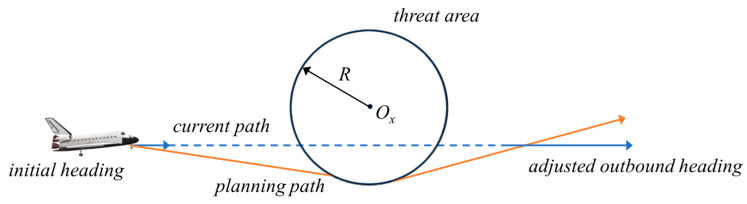
Threat area and path planning map.

**Figure 25:**
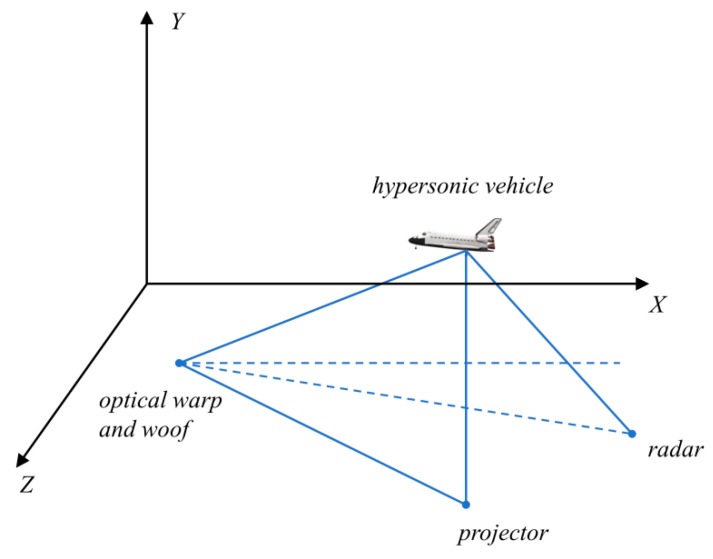
Vehicle versus radar position plot.

With the continuous development of weapons technology, the system in the field of military defense and control is being gradually improved. The traditional ballistic missile path may face the risk of being predicted or even intercepted, which is not safe. For the actual ballistic path optimization design problem, different tactical indicators often have different optimization objectives. Hypersonic cruise missiles fly extremely fast and can change their trajectory, thus greatly reducing the risk of interception. These characteristics make it possible to attack targets with very short warning times and at very high speeds. However, current research in this area is relatively small and has not achieved a major breakthrough. In this section, we look at cruise missile trajectories at hypersonic speeds, first considering only the following two conditions:

The hypersonic flight threat area and trajectory map are shown in Figure 24. The constrained region is shown in Figure 25, which shows the positional coordinates of the craft in relation to the radar. In this paper, the radar-centered range of 400 km is used as the solution space, and it is considered that the vertical distance between each defense unit is as far as possible, thus increasing the lateral distance of the interceptor missile.

Hypersonic missile trajectory modeling needs to satisfy certain conditions. Assuming that there are a total of *n* cubic curves, the curvature of the *i*-th curve is denoted as *p_i_*(*t*), 1≤i≤n. The length of the curve is denoted as $l_{i}$, and the derivative of the curvature is denoted as $d_{i}^{\prime }(t)$. The control fixed points are $B_{i,0},B_{i,1},B_{i,2},B_{i,3}$, respectively.

**Optimization Objective**: The length of the missile trajectory curve is the shortest and the curvature derivative is the largest.

**Limitations**: The range of feasible domains, continuity constraints, maximum curvature constraints.

**Decision Variable**: The control vertex.

Minimize

$$k_{1}\sum_{i=1}^{n}l_{i}+k_{2}\sum_{i=1}^{n}D_{i}(t).$$

subject to

$$D_{i}(t)=\min({d^{\prime }}_{i}(t)),\;0\leq d_{i}(t)\leq 1/R,\;i=1,2,\ldots ,n,\;j=0,1,2,3,\;l_{i}=\int_{0}^{1}\sqrt{{\left(p^{\prime }(t)\right)}^{2}+{\left(q^{\prime }(t)\right)}^{2}}dt,\;d_{i}(t)=\frac{p^{\prime }(t)q^{\prime \prime }(t)-q^{\prime }(t)p^{\prime \prime }(t)}{{\left({\left(p^{\prime }(t)\right)}^{2}+{\left(q^{\prime }(t)\right)}^{2}\right)}^{3/2}}.$$

where *k*_1_ and *k*_2_ are the weighting factor.

The above is a more complex minimization problem proposed in this paper. Next, CMRLCCOA is used to solve it for hypersonic missiles.

### 6.2. Solving the Model

CMRLCCOA, as well as KOA, TROA, BWO, AO, HBA, SWO, GMO, OMA, and GO, is applied to the hypersonic cruise ballistic optimization problem. Table 11 lists the optimal ballistic lengths solved by the 10 algorithms. Observing the table, it can be observed that the shortest length obtained by CMRLCCOA is 51.231801 km. This result is less than the results calculated by the others. This phenomenon indicates that CMRLCCOA performs better.

## 7. Summary and Outlook

In this paper, four strategies are used to improve COA, which leads to the proposed CMRLCCOA. First, in the initialization population phase, the coati population is initialized using the Sine chaotic mapping function to avoid population randomization. Second, a lens imaging reverse learning strategy is applied to renew the location of the coati population again. This strategy can expand the search space and enhance the quality of coati populations. Then, the Lévy flight strategy allows coatis to move over a wide range in the search space, reducing the iguana constraint. This method makes the algorithm better at finding a global optimum. Finally, the use of the crossover strategy reduces the search blind spots and improves the algorithm’s accuracy. Experiments are conducted in CEC2017 and CEC2019 test suites, where 50 and 100 dimensions are used in CEC2017. Lastly, the optimization results derived from CMRLCCOA are compared with COA, six new algorithms proposed in the last two years, four classical and well-recognized algorithms, and three enhanced algorithms. Then, we find that the newly proposed CMRLCCOA has better results and higher performance. In addition, CMRLCCOA is able to optimize to obtain better solutions to the three engineering problems. Finally, this paper also establishes a model of a hypersonic vehicle cruise ballistic problem. CMRLCCOA performs best in solving the hypersonic cruise ballistic trajectory optimization, reflecting the strong optimization capability and stability of CMRLCCOA.

To conclude, this study has strong scientific and practical value. The possible future work is as follows: Although the proposed CMRLCCOA has enhanced optimization capability and accelerated convergence speed, it still has areas of improvement in terms of computational complexity and computation time. CMRLCCOA will be further optimized for this problem in the follow-up work. In addition, we will continue to study on the basis of CMRLCCOA to obtain better solutions and apply it to address many complicated optimization problems, including route planning [87,88], image division problems [89], workshop scheduling [90], feature selection [91,92], shape optimization [76], and engineering optimization [93], and further expand the application field of intelligent algorithms.

## Figures and Tables

**Table 1:** Introduction to ten common chaotic mapping functions.

NO.	Function Name	Function Definition	Parameter
1	Sinusoidal [48]	$z_{k+1}=bz_{k}^{2}\sin(\pi \;z_{k})$	*b* = 2.3 and *z*_0_ = 0.7.
2	Logistic [49]	$z_{k+1}=bz_{k}(1-z_{k})$	*z_k_* is the *k*th chaotic number, and *z*_0_∈(0, 1).
3	Tent [50]	$z_{k+1}=\left\{\begin{array}{l} \frac{z_{k}}{\alpha },z_{k}\in (0,\alpha ]\\ \frac{1-z_{k}}{1-\alpha },z_{k}\in (z_{k},1] \end{array}\right.$	-
4	Gauss/Mouse [51]	$z_{k+1}=\left\{\begin{array}{l} 0,z_{k}=0\\ \mathrm{mod}(\frac{\mu }{z_{k}},1),otherwise \end{array}\right.$	Generates chaotic sequences in (0, 1).
5	Circle [52]	$z_{k+1}=z_{k}+b-\mathrm{mod}(\frac{a}{2\pi }\sin(2\pi z_{k}),1)$	*a =* 2.2 and *b =* 0.5.
6	Chebyshev [53]	$z_{k+1}=\cos(k\cos^{-1}(z_{k}))$	-
7	Singer [54]	$z_{k+1}=\mu (7.86z_{k}-23.31z_{k}^{2}+28.75z_{k}^{3}-13.30z_{k}^{4})$	*µ* is set between 0.9 and 1.08.
8	Bernoulli [55]	$z_{k+1}=\left\{\begin{array}{l} \frac{z_{k}}{1-\lambda },z_{k}\in (0,1-\lambda ]\\ \frac{z_{k}-1+\lambda }{\lambda },z_{k}\in (1-\lambda ,1) \end{array}\right.$	-
9	ICMIC [56]	$z_{k+1}=\sin\left(\frac{a}{z_{k}}\right)$	*a*∈(0, ∞).
10	Piecewise [57]	$x_{k+1}=\left\{\begin{array}{l} z_{k}/q,z_{k}\in [0,q)\\ \left(z_{k}-q)/(0.5-q),z_{k}\in [q,\;0.5\right)\\ \left(1-n-z_{k})/(0.5-n),z_{k}\in [0.5,\;1-n\right)\\ \left(1-z_{k})/n,z_{k}\in [1-n,\;1\right) \end{array}\right.$	*n*∈(0, 0.5) and *z_k_*∈[0, 1].

**Table 2:** Algorithm-related information.

Algorithm	Year	Parameter Name	Value
PSO	1995	Inertia weight	Decreasing linearly from 0.9 to 0.1
		Velocity range	0.1 times the size of the variable
		Cognitive and social factors	*c*1 = 2, *c*2 = 2
DE	1995	Scaling factor	0.5
		Crossover probability	0.5
SA	1953	-	-
ABC	2005	Limit	20
KOA	2023	Velocity	*Tc* = 3, *M*_0_ = 0.1, λ = 15
SWO	2023	Hunting and nesting weight	0.5
GMO	2023	Dual-fitness index	*α =* 0.05*, Pa_2_ =* 0.2*, Prb =* 0.2
OMA	2023	Space	0.55
TROA	2023	Hunting success rate	[0.1,.1]
GO	2024	Stone weight	*p*1 = 5, *p*2 = 0.001, *p*3 = 0.3
IGWO	2021	*α*	Decreases linearly from 2 to 0
EWOA	2023	a	Decreased from 2 to 0
		b	2

**Table 3:** Results of chaotic mapping functions in the CEC2019 test set.

Function	Chaos Mapping, Rank									
	Tent		Logistic		Cubic		Chebyshev		Piecewise	
CEC01	1	1	1	1	1	1	1	1	1	1
CEC02	4.8343428	3	4.9086048	4	4.7536217	1	4.9480054	9	4.9127265	5
CEC03	4.5113105	9	3.6855279	2	4.4167636	8	4.0965744	6	4.5839437	10
CEC04	21.433848	7	30.186825	10	24.332956	8	16.31391	1	27.812378	9
CEC05	1.1617451	5	1.1431999	1	1.1515950	3	1.1631781	6	1.194418	9
CEC06	3.3690697	4	3.3841554	5	3.2145844	3	3.6269374	9	3.4008807	6
CEC07	732.95347	9	725.80671	8	614.88261	4	584.74687	2	618.38094	6
CEC08	2721.4771	2	2647.8950	7	2851.1804	10	2604.0760	5	2792.7936	8
CEC09	3.6744951	5	3.8464906	6	3.9101588	3	3.7696891	8	3.8590904	9
CEC10	1.1916796	1	1.2024768	8	1.1872587	9	1.2116937	5	1.2209108	7
Ave rank	4.6		5.2		5		5.2		7	
Final rank	4		6		5		6		10	
Function	Chaos mapping, Rank									
	Sinusoidal		Sine		ICMIC		Circle		Bernoulli	
CEC01	1	1	1	1	1	1	1	1	1	1
CEC02	4.9393187	7	4.9476647	8	4.9675991	10	4.9228798	6	4.7674341	2
CEC03	4.1652904	7	3.6007047	1	3.8968772	4	3.9812347	5	3.7443864	3
CEC04	19.948643	5	16.530138	2	19.427083	4	20.471053	6	18.128766	3
CEC05	1.1669640	7	1.1614536	4	1.1923656	8	1.1509138	2	1.200149	10
CEC06	3.6426736	10	3.4905846	8	3.4168389	7	3.1405742	2	2.8830131	1
CEC07	813.11549	10	580.91894	1	615.26845	5	688.45524	7	604.38442	3
CEC08	2734.7201	6	2530.4876	1	2646.3779	9	2686.5244	3	2650.1646	4
CEC09	3.7708771	10	3.5869369	1	3.8648624	7	3.7574923	2	3.7603243	4
CEC10	1.2469245	6	1.1435522	4	1.2066848	10	1.1592935	2	1.1908522	3
Total rank	6.9		3.1		6.5		3.6		3.4	
Final rank	9		1		8		3		2	

**Table 4:** Comparison results of CMRLCCOA and different algorithms in CEC2019.

Function	Index	COA	CMRLCCOA	KOA	SWO	GMO	OMA	TROA	GO	PSO	DE	SA	ABC	ISSA	IGWO	EWOA
cec01	Ave	1	1	1.84E+08	1.48E+07	1	2.84E+05	1.67E+09	1.69E+08	1	5.20E+06	1	1.05E+07	1	5.79E+04	2.37E+06
	Worst	1	1	4.54E+08	1.64E+08	1	1122748	3.12E+09	2.92E+08	1	11058720	1	23234725	1	314276	13879628
	Best	1	1	2.44E+07	9.39E+01	1	3.36E+04	3.74E+08	2.25E+07	1	5.33E+05	1	2.90E+06	1	1.23E+01	5.95E+04
	Std	0	0	9.53E+07	3.73E+07	9.92E-08	2.48E+05	8.49E+08	7.31E+07	0	3.20E+06	0	5.06E+06	0	8.21E+04	3.05E+06
	Rank	1	1	14	12	6	8	15	13	1	10	1	11	1	7	9
cec02	Ave	5	4.818	12317.590	861.263	4.901	294.950	30233.500	9853.688	4.524	2666.823	4679.524	5229.067	4.254	334.713	2764.978
	Worst	5	5.000	16901.760	4888.710	5.016	1082.046	48225.370	15077.270	4.767	4079.375	8195.320	6785.122	4.374	520.294	6792.063
	Best	5	4.227	8797.222	6.154	4.484	103.766	19285.510	6266.771	4.313	1603.955	1354.395	3788.566	4.217	184.893	494.261
	Std	1.04E-06	0.262	1984.790	1278.936	0.162	201.267	7041.787	2262.994	0.150	716.899	2066.515	747.501	0.034	88.850	1925.375
	Rank	5	3	14	8	4	6	15	13	2	9	11	12	1	7	10
cec03	Ave	4.622	2.106	11.433	10.332	1.993	3.473	12.699	11.374	2.815	5.432	6.197	10.087	2.278	1.864	6.742
	Worst	5.475	5.512	12.168	11.634	3.475	4.739	13.278	12.170	4.564	6.231	10.712	10.935	7.686	7.712	9.710
	Best	2.943	1.317	10.743	6.714	1.000	2.343	11.973	9.492	1.413	3.979	1.409	8.684	1.002	1.001	3.237
	Std	0.661	0.809	0.384	1.179	0.833	0.823	0.255	0.544	0.879	0.675	2.878	0.546	1.420	1.535	1.788
	Rank	7	3	14	12	2	6	15	13	5	8	9	11	4	1	10
cec04	Ave	83.126	11.211	81.242	81.491	25.163	24.406	155.292	74.851	98.306	13.142	23.918	36.051	28.804	12.690	30.922
	Worst	110.190	27.918	93.461	103.081	66.180	43.391	202.658	93.995	98.506	17.302	44.599	43.256	50.679	23.203	62.484
	Best	60.535	3.992	65.997	62.612	5.975	14.378	102.226	50.088	97.510	5.662	7.965	26.430	5.975	4.008	13.934
	Std	14.114	5.133	8.691	10.972	14.740	7.680	26.491	10.343	0.398	2.749	9.215	3.976	10.740	6.509	12.803
	Rank	13	1	11	12	6	5	15	10	14	3	4	9	7	2	8
cec05	Ave	88.662	1.209	32.158	28.849	1.226	1.232	201.010	25.001	6.406	1.349	1.213	1.485	1.138	1.606	2.084
	Worst	143.853	1.345	55.645	68.663	1.885	1.521	281.706	51.233	14.646	1.421	1.608	1.637	1.426	1.793	10.596
	Best	34.877	1.046	16.528	10.402	1.039	1.041	60.285	9.520	2.341	1.062	1.012	1.306	1.034	1.423	1.030
	Std	29.671	0.103	10.825	15.632	0.226	0.113	64.150	9.822	3.244	0.042	0.165	0.085	0.093	0.093	2.796
	Rank	14	2	13	12	4	5	15	11	10	6	3	7	1	8	9
cec06	Ave	10.416	3.083	11.411	10.418	1.612	1.980	15.880	10.706	16.019	2.226	6.393	3.298	4.713	1.838	5.789
	Worst	12.364	5.473	13.788	12.757	4.735	4.450	17.642	12.658	16.858	2.752	9.150	5.090	7.272	3.322	10.030
	Best	8.101	1.915	9.318	6.511	1.013	1.162	11.850	8.652	14.849	1.745	3.805	1.568	2.555	1.138	2.122
	Std	1.128	0.973	1.219	1.247	0.915	0.751	1.312	1.115	0.586	0.275	1.533	0.863	1.360	0.457	2.247
	Rank	10	5	13	11	1	3	14	12	15	4	9	6	7	2	8
cec07	Ave	1888.543	630.452	2214.700	1984.343	1127.429	1488.903	2920.239	2057.648	1966.054	692.845	720.485	1429.335	1002.165	695.429	966.935
	Worst	2213.910	998.426	2555.673	2365.470	2086.663	1744.456	3437.083	2347.304	2156.438	919.701	1130.655	1711.483	1582.198	1431.710	1526.280
	Best	1364.789	271.372	1825.462	1408.175	357.282	1256.515	2219.054	1619.865	1818.921	411.468	245.729	1155.446	519.223	5.542	125.939
	Std	238.431	203.125	183.251	250.080	521.197	144.181	328.974	210.137	111.793	132.633	229.616	164.750	253.134	473.452	318.565
	Rank	10	1	14	12	7	9	15	13	11	2	4	8	6	3	5
cec08	Ave	4.839	3.174	5.202	5.084	3.991	4.055	5.601	5.110	5.517	3.794	4.279	4.478	4.293	3.235	4.418
	Worst	5.055	4.638	5.476	5.352	4.931	4.333	5.874	5.475	5.560	4.034	5.203	4.777	5.000	3.865	4.932
	Best	4.149	3.275	4.997	4.816	3.133	3.342	5.147	4.822	5.493	3.371	3.502	4.104	3.455	2.059	3.825
	Std	0.227	0.368	0.140	0.147	0.510	0.239	0.179	0.168	0.022	0.198	0.434	0.177	0.398	0.562	0.293
	Rank	10	1	13	11	4	5	15	12	14	3	6	9	7	2	8
cec09	Ave	3.726	1.209	2.528	2.312	1.095	1.495	6.666	2.264	1.234	1.275	1.486	1.277	1.330	1.216	1.435
	Worst	4.445	1.385	3.225	3.296	1.208	1.829	8.271	3.043	1.377	1.337	1.904	1.362	1.475	1.297	1.723
	Best	2.779	1.056	1.925	1.544	1.045	1.251	5.311	1.601	1.083	1.185	1.058	1.169	1.144	1.081	1.196
	Std	0.488	0.084	0.313	0.453	0.038	0.146	0.733	0.401	0.098	0.037	0.225	0.056	0.108	0.047	0.156
	Rank	14	2	13	12	1	10	15	11	4	5	9	6	7	3	8
cec10	Ave	21.455	21.017	21.788	21.635	19.365	21.381	22.101	21.737	20.985	20.934	21.019	21.452	21.368	21.434	21.462
	Worst	21.624	21.464	21.992	21.832	21.601	21.566	22.367	21.978	20.987	21.191	21.115	21.620	21.592	21.577	21.628
	Best	21.205	21.145	21.413	21.373	1.001	20.591	21.709	21.464	20.983	18.057	21.000	21.253	21.027	21.263	21.185
	Std	0.121	0.082	0.140	0.143	6.123	0.201	0.193	0.126	0.001	0.705	0.032	0.098	0.162	0.088	0.106
	Rank	10	4	14	12	1	7	15	13	3	2	5	9	6	8	11
Mean Rank		9.4	2.3	13.3	11.4	3.6	6.4	14.9	12.1	7.9	5.2	6.1	8.8	4.7	4.3	8.6
Final Ranking		11	1	14	12	2	7	15	13	8	5	6	10	4	3	9

**Table 5:** Significance of CMRLCCOA and different algorithms in CEC2019.

Function	COA	KOA	SWO	GMO	OMA	TROA	GO	PSO	DE	SA	ABC	ISSA	IGWO	EWOA
cec01	NaN/=	8.01E-09/-	6.08E-08/-	2.99E-08/-	8.01E-09/-	8.01E-09/-	6.08E-01/-	NaN/=	6.51E-08/-	NaN/=	8.01E-09/-	NaN/=	8.01E-09/-	7.93E-09/-
cec02	1.57E-08/-	6.80E-08/-	9.23E-06/-	1.33E-02/-	6.80E-08/-	7.33E-07/-	6.80E-08/-	7.58E-04/+	9.21E-08/-	6.33E-08/-	3.76E-06/-	7.95E-07/+	6.31E-08/-	6.80E-08/-
cec03	3.85E-02/-	7.99E-08/-	6.80E-08/-	7.95E-07/+	1.93E-02/-	6.80E-08/-	6.31E-07/-	2.22E-04/-	1.81E-05/-	1.79E-02/-	6.80E-08/-	7.58E-06/-	6.67E-06/+	9.75E-06/-
cec04	6.80E-08/-	6.04E-09/-	3.88E-09/-	4.39E-02/-	1.01E-02/-	9.38E-06/-	9.03E-06/-	6.80E-08/-	8.59E-02/=	9.05E-03/-	7.92E-09/-	1.79E-04/-	6.04E-03/-	1.61E-04/-
cec05	7.32E-07/-	6.80E-08/-	7.90E-08/-	3.10E-01/=	5.08E-01/=	3.20E-05/-	5.30E-06/-	6.80E-08/-	1.33E-01/=	6.95E-01/=	5.23E-07/-	3.37E-02/+	9.11E-09/-	2.62E-01/=
cec06	6.80E-08/-	4.26E-07/-	6.80E-08/-	3.99E-06/+	1.25E-05/+	6.80E-08/-	7.69E-07/-	9.60E-04/-	1.81E-05/+	2.32E-05/-	4.41E-01/-	1.79E-02/-	2.56E-07/+	2.561E-03/-
cec07	3.68E-08/-	7.30E-08/-	6.80E-08/-	3.97E-03/-	6.80E-08/-	6.80E-08/-	9.01E-06/-	7.31E-06/-	2.50E-01/=	2.62E-01/=	2.50E-07/-	8.29E-05/-	8.18E-01/=	3.75E-04/-
cec08	7.88E-05/-	6.80E-08/-	9.10E-06/-	3.99E-06/-	1.25E-05/-	6.36E-09/-	6.80E-08/-	6.80E-08/-	1.81E-05/-	2.36E-06/-	4.41E-01/=	1.79E-02/-	2.56E-07/-	2.56E-03/-
cec09	8.21E-08/-	6.80E-08/-	6.80E-08/-	1.81E-05/+	6.01E-07/-	6.80E-08/-	3.67E-04/-	2.98E-01/=	2.80E-03/-	1.61E-04/-	6.04E-03/-	9.21E-04/-	2.39E-02/-	1.41E-05/-
cec10	9.05E-03/-	1.23E-07/-	1.38E-06/-	1.26E-01/=	7.64E-02/-	6.80E-08/-	6.80E-08/-	5.33E-05/+	2.56E-07/+	6.80E-08/-	3.06E-03/-	5.08E-01/=	1.06E-02/-	1.95E-03/-
+/=/-	0/1/9	0/0/10	0/0/10	3/2/5	1/1/8	0/0/10	0/0/10	2/2/6	2/3/5	0/3/7	0/1/9	2/2/6	2/1/7	0/1/9

**Table 6:** The discrete value of the standard modulus.

*x*3: Standard Modulus (mm)						
0.1	0.12	0.15	0.2	0.25	0.3	0.4
0.5	0.6	0.8	0.1	1.25	1.5	2
2.5	3	4	5	6	8	10
12	16	20	25	32	40	50

**Table 7:** The results of the SSCGR problem.

Algorithms	*x*_1_	*x*_1_	*x*_1_	*x*_1_	*x*_1_	*x*_1_
CMRLCCOA	84.3521	164.5389	12	76	96	15
KOA	68.31117	163.3201	12	61	87	19
TROA	127.9832	236.9841	6	100	138	23
BWO	125.2962	242.9624	10	77	72	15
AO	52.0544	150.0000	8	68	84	19
HBA	59.0237	150.0234	8	67	83	21
SWO	112.3465	265.799	6	108	148	24
GMO	125.6832	234.0342	5	107	140	27
OMA	120.4782	243.8961	6	100	98	24
GO	120.9341	230.4218	6	108	138	25

**Table 8:** The solution quality of SSCGR problem.

Algorithms	Best Cost	Worse Cost	Average Cost	Standard Deviation
CMRLCCOA	9,753,030	19,553,655	15,358,330	258,799.75
KOA	11,703,116	49,804,525	23,770,950	9,668,408.43
TROA	11,020,774	37,884,551	20,846,936	6,707,838.46
BWO	14,902,880.2	17,193,421.69	15,818,208.26	544,502.38
AO	15,235,791.91	16,184,369.46	15,671,961.51	255,292.88
HBA	15,525,037.23	16,184,369.46	15,986,219	282,022.135
SWO	19,478,312.31	28,378,921.72	24,589,213.91	5,423,637.31
GMO	20,786,321.63	34,762,811.82	28,970,643.84	6,272,892.32
OMA	28,876,731.35	34,678,891.78	31,328,901.12	256,893.21
GO	26,895,531.98	36,755,467.90	31,548,903.82	4,983,221.34

**Table 9:** Numerical results of ten algorithms for the WBD.

Element	CMRLCCOA	KOA	TROA	BWO	AO	HBA	SWO	GMO	OMA	GO
*x*_1_	0.634	0.163	0.205	0.204	0.156	0.723	0.621	0.169	0.483	0.341
*x*_2_	4.211	4.981	3.572	3.721	5.242	1.502	3.411	4.822	3.274	2.592
*x*_3_	6.802	9.164	9.824	9.311	8.968	5.368	5.391	9.231	8.021	7.608
*x*_4_	0.633	0.246	0.214	0.287	0.219	0.642	0.511	0.205	0.425	0.328
Best	1.660	1.664	1.661	1.663	1.747	2.011	1.683	1.662	1.673	1.691
Worse	1.671	2.130	1.683	2.173	2.162	4.702	6.326	2.750	2.238	2.691
Mean	1.665	1.932	1.674	1.801	1.944	3.164	3.890	2.137	1.902	2.185
Std	0.006	0.132	0.008	0.121	0.133	0.783	1.311	0.185	0.133	0.170

**Table 10:** Statistical results of cantilever beam design issues.

Element	CMRLCCOA	KOA	TROA	BWO	AO	HBA	SWO	GMO	OMA	GO
*x*_1_	5.970619	6.340312	5.934021	6.094141	5.952939	6.023157	6.093914	5.873219	6.013591	5.909183
*x*_2_	5.271230	5.329041	5.312115	5.245089	5.279312	5.372044	5.172349	5.309218	5.305521	5.328713
*x*_3_	4.463102	4.502875	4.476823	4.454137	4.47316	4.780241	4.768092	4.457552	4.432802	4.795219
*x*_4_	3.476491	3.592133	3.508861	3.425591	3.469623	3.542192	3.523891	3.492033	3.509931	3.480216
*x_5_*	2.137348	2.160322	2.436287	2.102532	2.149688	2.023797	2.153571	2.437761	2.189233	2.039822
Best	13.302191	16.433288	14.306442	13.32191	13.317692	13.347884	13.863016	13.712833	13.690375	13.926679
Worse	3.313796	24.306681	27.683391	13.396380	13.329414	14.216902	13.926871	16.086629	14.283347	19.37228
Mean	13.308977	20.499271	19.903173	13.358301	13.322805	13.983346	13.890766	14.03799	13.998273	15.349741
Std	3.16E-05	3.8891	4.0399	1.98E-02	2.93E-03	2.57E-02	1.79E-02	9.91E-01	3.88E-02	2.67E-01

**Table 11:** Path lengths of CMRLCCOA and other algorithms.

Element	CMRLCCOA	KOA	TROA	BWO	AO	HBA	SWO	GMO	OMA	GO
Length/km	51.231801	57.228763	58.183246	51.243317	51.375423	53.902461	55.320411	51.410963	53.801934	57.944201

## Data Availability

All data generated or analyzed during the study are included in this published article.

## Appendix A. Results of the CEC2017 Test Set

See Table A1, Table A2, Table A3 and Table A4.

**Table A1:** Optimization results of CMRLCCOA algorithm with 14 algorithms in cec2017 function when *dim* = 50.

Function	Index	Algorithms														
		COA	CMRLCCOA	KOA	SWO	GMO	OMA	TROA	GO	PSO	DE	SA	ABC	ISSA	IGWO	EWOA
CEC01	Ave	1.08E+11	22,777,249	1.18E+11	1.07E+11	43,547.92	8.9E+09	2.67E+11	1.49E+11	1.23E+10	1.28E+08	22,115.01	4.19E+09	4,589,023	3.26E+08	1.13E+10
	Std	2.56E+09	7,420,778	1.47E+10	1.26E+10	20,964.4	2.01E+09	8.81E+09	1.09E+10	4.49E+09	46,273,671	17,616.93	3E+08	3,000,020	2.78E+08	8.81E+09
	Rank	12	4	13	11	2	8	15	14	10	5	1	7	3	6	9
CEC03	Ave	193,393.7	83,357.22	495,184.8	374,612.9	151,513.8	168,988.4	1.63E+10	14,125,521	108,651.2	261,878.2	427,856.9	333,521.1	258,155.3	61,505.58	324,586.3
	Std	16,512.06	13,517.11	120,262.1	55,484.44	11,447.75	22,600.47	3.2E+10	25,904,509	16,905.62	23,397.86	109,269.6	45,840.02	52,501	7858.062	73,677.19
	Rank	6	2	13	11	4	5	15	14	3	8	12	10	7	1	9
CEC04	Ave	37,169.12	666.11843	29,217.66	32,616.67	595.0011	1957.124	116,582.1	50,507.09	3696.137	862.5515	658.4734	1263.84	655.8	659.9922	2276.58
	Std	8525.985	50.203537	9385.45	4779.994	47.57367	613.9945	12,298.04	7394.986	856.2404	17.60447	79.87179	91.04166	85.99321	25.16279	1473.755
	Rank	13	5	11	12	1	8	15	14	10	6	3	7	2	4	9
CEC05	Ave	1193.308	739.7449	1290.262	1289.723	678.3483	850.7566	1683.433	1356.928	888.8936	904.5467	783.4538	1010.374	872.9973	705.418	926.5937
	Std	22.23096	34.83147	16.77681	42.89903	43.8473	33.98982	64.33351	25.00527	8.49458	18.80467	25.24603	16.72415	33.29331	82.20668	47.40865
	Rank	11	3	13	12	1	5	15	14	7	8	4	10	6	2	9
CEC06	Ave	697.2991	652.73418	708.4708	707.0367	617.3871	638.1543	746.2198	714.9182	674.0721	602.7132	600.5653	623.6647	659.8166	603.5628	656.9779
	Std	4.636789	9.3784893	3.542636	8.981565	5.473167	2.657291	6.883185	4.726849	2.803705	0.159644	0.360944	2.518193	11.04104	0.565964	7.909236
	Rank	11	7	13	12	4	6	15	14	10	2	1	5	9	3	8
CEC07	Ave	2034.219	1254.761	3043.196	2108.583	900.3868	1681.005	5910.989	3364.926	1819.316	1174.162	1083.525	1413.702	1528.194	1014.83	1427.293
	Std	43.61301	118.2879	371.1122	58.89466	25.55611	168.9367	403.1108	293.7753	17.72496	15.6616	49.23108	12.65898	150.4762	109.4659	312.1561
	Rank	11	5	13	12	1	9	15	14	10	4	3	6	8	2	7
CEC08	Ave	1477.196	1144.4651	1614.532	1549.343	1020.067	1203.821	1986.525	1682.505	1240.873	1192.076	1057.334	1307.304	1145.338	982.4962	1192.125
	Std	13.15204	65.467167	47.45637	32.80135	147.442	63.90452	26.18005	27.834	6.61208	13.27879	62.73983	45.10759	29.54384	30.64892	55.9351
	Rank	11	4	13	12	2	8	15	14	9	6	3	10	5	1	7
CEC09	Ave	36,375.47	3960.819	55,732.01	49,408.77	2846.232	15,906.27	108,071.8	67,110.53	17,183.96	11,831.44	21,525.81	12,520.32	12,959.32	2920.979	23,925.96
	Std	2849.587	1075.734	7913.843	2455.993	2283.157	1504.345	8244.483	8710.391	917.6917	920.0699	2877.513	1540.828	676.074	849.9576	15,603.63
	Rank	11	3	13	12	1	7	15	14	8	4	9	5	6	2	10
CEC10	Ave	15,101.72	8546.989	16,203.35	15,835.74	5414.964	14,919.44	17,242.51	16,910.83	9302.555	12,483.82	7057.742	15,084.94	7065.536	13,192.26	9308.447
	Std	221.4496	682.51606	399.6127	841.2409	897.9448	331.3749	1234.191	233.4546	331.255	400.1101	417.6019	223.1407	474.6596	2732.186	626.4859
	Rank	11	4	13	12	1	9	15	14	5	7	2	10	3	8	6
CEC11	Ave	24,864.66	1361.044	44,046.72	31,941.3	1587.692	2697.973	109,948.7	44,945.56	2221.056	6490.606	5980.947	8613.898	1751.453	1477.223	3489.805
	Std	1508.507	25.92436	10,908.1	6367.875	120.0267	548.0429	28,355.74	9232.634	186.8323	1611.709	5798.111	214.1184	75.62217	112.0471	1468.077
	Rank	11	1	13	12	3	6	15	14	5	9	8	10	4	2	7
CEC12	Ave	6.72E+10	20,936,519	5E+10	4.72E+10	55,244,111	5.16E+08	1.38E+11	5.79E+10	2.8E+09	2.49E+08	22,295,145	5.49E+08	19,124,122	51,274,629	2E+09
	Std	1.15E+10	4,209,179.7	1.11E+10	9.29E+09	34,524,448	2.56E+08	1.9E+10	8.63E+09	9.69E+08	37,525,785	7,007,320	1.42E+08	5,497,266	31,451,961	1.35E+09
	Rank	14	2	12	11	5	7	15	13	10	6	3	8	1	4	9
CEC13	Ave	4.13E+10	29,603.37	1.69E+10	2.28E+10	69,344.11	10,428,566	7.66E+10	2.53E+10	4.85E+08	9,842,121	13,215.19	15,946.13	29,501.78	731,335.7	7.33E+08
	Std	4.45E+09	10,333.93	1.01E+09	8.46E+09	30,601.48	8,246,186	2.85E+10	6.37E+09	4.08E+08	3,460,376	8034.778	7434.498	9961.064	315,648.8	1.35E+09
	Rank	14	4	11	12	5	8	15	13	9	7	1	2	3	6	10
CEC14	Ave	65,470,166	229,560.17	29,239,168	72,680,621	279,850.6	225,788.2	3.19E+08	1.09E+08	1,936,955	2,688,963	5,564,561	3,131,591	657,740.9	112,276.5	1,570,218
	Std	81,051,780	107,000.38	5,317,825	66,025,071	199,602.7	240,768.8	1.3E+08	74,868,291	2,057,292	828,156	4,939,227	916,533.5	212,279.2	60,721.49	1,164,916
	Rank	12	3	11	13	4	2	15	14	7	8	10	9	5	1	6
CEC15	Ave	9.89E+09	16,700.68	4.89E+09	4.46E+09	17,082.37	33,754.76	2.56E+10	9.46E+09	49,965.49	1,548,365	11,555.33	17,365.23	15,979.73	210,972.8	28,621,778
	Std	2.3E+09	9719.672	1.26E+09	1.66E+09	4809.354	48,172.26	6.24E+09	2.33E+09	65,045.48	551,610.4	7479.573	9723.996	7150.865	118,015.6	34,812,203
	Rank	14	3	12	11	4	6	15	13	7	9	1	5	2	8	10
CEC16	Ave	9099.678	3207.398	7618.124	8901.292	3039.643	4477.407	15,601.72	8329.691	4518.336	4322.064	3834.053	5282.878	3208.344	2878.653	4429.806
	Std	298.344	375.81309	245.3604	436.3029	388.9831	188.797	1509.998	673.5675	221.828	187.1573	729.1978	214.755	233.0231	444.956	352.1305
	Rank	14	3	11	13	2	8	15	12	9	6	5	10	4	1	7
CEC17	Ave	8729.022	2845.358	12,330.74	6696.481	2718.79	3631.466	2,322,642	46,408.27	3766.865	3443.919	2916.01	3996.468	3118.238	2641.981	3889.49
	Std	4081.734	248.9313	6270.508	323.4054	216.5457	332.3307	2,466,579	54,011.65	236.5153	208.5305	133.9688	116.1851	226.0531	619.8051	310.2085
	Rank	12	3	13	11	2	7	15	14	8	6	4	10	5	1	9
CEC18	Ave	2.38E+08	1,234,407.4	2.23E+08	1.17E+08	1,321,256	1,231,943	1.55E+09	3.11E+08	1,271,874	8,839,026	10,469,508	22,416,209	2,581,265	885,141.6	6,242,625
	Std	1.26E+08	1,161,304.1	1.14E+08	31,758,781	468,364.3	1,091,914	5.57E+08	1.67E+08	254,059	2,981,783	6,952,494	5,835,715	1,260,645	444,424.6	4,106,505
	Rank	13	3	12	11	5	2	15	14	4	8	9	10	6	1	7
CEC19	Ave	3.49E+09	43,514.24	1.39E+09	2.72E+09	1,328,314	30,601.03	1.64E+10	4.89E+09	849,356.3	632,801.6	21,134.47	18,793.35	22,011.91	101,738.8	1,202,988
	Std	1.33E+09	9171.384	4.54E+08	9.39E+08	1,184,440	8086.937	2.96E+09	1.84E+09	797,817.2	164,401.3	14,463.14	7799.813	14,582.63	34,515.4	2,066,650
	Rank	13	5	11	12	10	4	15	14	8	7	2	1	3	6	9
CEC20	Ave	4264.671	3176.6481	5037.649	4958.044	2929.479	3959.729	5635.665	4924.366	3911.16	3391.586	3363.697	4133.166	3117.517	3200.931	3468.399
	Std	217.298	166.08	231.7725	237.1699	433.6476	129.4059	189.2257	213.2764	132.3606	316.4075	412.272	130.6882	280.4358	668.7847	206.062
	Rank	11	3	14	13	1	9	15	12	8	6	5	10	2	4	7
CEC21	Ave	3231.155	2344.293	3166.854	3110.984	2467.589	2632.248	3490.363	3178.499	2983.725	2691.148	2583.096	2790.708	2649.308	2479.574	2653.103
	Std	45.04548	51.00179	63.29145	62.67646	32.91107	60.53255	106.4844	45.90163	49.42571	20.37949	23.93868	22.74097	109.809	23.21851	75.09771
	Rank	14	1	12	11	2	5	15	13	10	8	4	9	6	3	7
CEC22	Ave	16,454.59	10,865.898	18,037.24	17,890.48	8004.233	16,180.67	19,580.2	18,154.02	14,080.81	14,360.75	8665.501	16,575.73	11,106.81	13,692.32	10,633.68
	Std	413.1295	467.39056	376.7972	493.6783	2890.267	715.3968	574.2815	469.1817	213.242	322.9298	738.4887	379.5504	941.0086	4692.072	785.1567
	Rank	10	4	13	12	1	9	15	14	7	8	2	11	5	6	3
CEC23	Ave	4591.849	3001.786	3957.705	4288.638	3931.111	3456.039	5008.414	4025.427	5706.731	3146.138	3003.78	3215.223	3357.584	2980.28	3247.391
	Std	89.95229	54.11106	53.852	100.1697	81.06274	96.068	319.3221	49.19432	179.572	6.093371	87.61949	10.52026	350.6283	120.8443	115.2883
	Rank	13	2	10	12	9	8	14	11	15	4	3	5	7	1	6
CEC24	Ave	4847.797	3231.4568	4115.103	4862.816	3090.177	3408.62	5764.142	4135.503	3806.828	3380.324	3281.577	3349.475	3393.734	3098.505	3326.246
	Std	212.537	51.47082	109.419	337.2296	73.29847	39.81138	385.9678	112.9797	107.01	8.721637	48.63015	10.76343	178.0664	105.0975	12.47433
	Rank	13	3	11	14	1	9	15	12	10	7	4	6	8	2	5
CEC25	Ave	15,498.68	3148.276	17,123.51	15,205.47	3062.584	4548.281	57,542.68	28,931.78	4520.973	3282.677	3078.34	3797.379	3174.841	3203.052	3386.577
	Std	899.3804	31.63694	3609.818	1089.657	23.07329	510.3022	6522.157	3238.07	201.4541	34.31462	10.85326	80.88986	43.57991	45.31785	309.0026
	Rank	12	3	13	11	1	10	15	14	9	6	2	8	4	5	7
CEC26	Ave	16,669.95	6579.2521	18,986.31	17,692.56	2900.934	10,710.99	32,282.28	19,108.84	12,965.48	7752.529	7172.061	8627.301	8630.337	6004.097	8398.559
	Std	767.8479	747.62355	1052.36	869.9465	0.127582	996.7537	3493.074	581.5774	496.3631	166.1123	729.2199	147.012	3003.13	779.4425	1175.369
	Rank	11	3	13	12	1	9	15	14	10	5	4	7	8	2	6
CEC27	Ave	6725.142	3200.012	5903.513	6138.577	3428.48	3829.855	9553.585	5847.802	11871.15	3729.927	3565.047	3591.646	3518.086	3315.919	3930.156
	Std	508.8131	0.000107	262.9817	553.7433	29.68044	95.57017	916.492	516.5143	522.0628	30.22302	100.1004	10.31284	93.57749	26.93388	202.9049
	Rank	13	1	11	12	3	8	14	10	15	7	5	6	4	2	9
CEC28	Ave	12,383.48	3300.012	14,706.42	11,444.54	3341.592	4973.66	24,354.67	14,033.13	5608.795	3668.433	3353.469	5002.39	3404.716	3570.996	4836.268
	Std	576.9375	9.85E-05	1124.724	1487.904	24.33589	323.4519	3063.77	804.8052	515.085	79.06832	28.3977	301.4169	25.25116	95.67398	1568.961
	Rank	12	1	14	11	2	8	15	13	10	6	3	9	4	5	7
CEC29	Ave	82,826.59	4272.29	19,484.39	30,984.81	4510.673	6019.704	3,041,045	128,315	8518.909	4863.249	4556.345	5851.252	4557.7	4330.394	5326.608
	Std	35,551.16	120.8293	5203.725	14,718.85	132.2595	575.5447	2,595,774	52,579.9	571.9604	185.5084	329.6816	292.1598	300.2586	337.9723	623.0801
	Rank	13	1	11	12	3	9	15	14	10	6	4	8	5	2	7
CEC30	Mean	4.61E+09	308,753.48	4.45E+09	3.99E+09	94,377,063	24,692,618	1.73E+10	5.89E+09	3.21E+08	23,709,127	1,689,550	43,351,659	3,526,259	24,012,726	1.25E+08
	Std	1.9E+09	179,270.61	1.84E+09	1.38E+09	18,863,257	9,651,455	2.23E+09	1.11E+09	1.56E+08	5,350,374	417,644.7	13,203,141	855,339.4	4,668,925	1.74E+08
	Rank	13	1	12	11	8	6	15	14	10	4	2	7	3	5	9
Mean Rank		12.0345	3	12.2414	11.8276	3.0690	7.0690	14.9310	13.3793	8.7241	6.3103	4.1034	7.6207	4.7586	3.3103	7.6207
Final Rank		12	1	13	11	2	7	15	14	10	6	4	8	5	3	8

**Table A2:** Optimization results of CMRLCCOA algorithm with 14 algorithms in CEC2017 function when *dim* = 100.

Function	Index	Algorithms														
		COA	CMRLCCOA	KOA	SWO	GMO	OMA	TROA	GO	PSO	DE	SA	ABC	ISSA	IGWO	EWOA
CEC01	Ave	2.73E+11	4.76E+08	3.05E+11	2.5E+11	7,628,116	9.6E+10	6.39E+11	3.94E+11	8.41E+10	3.12E+09	10,307,943	8.24E+10	2.43E+09	1.76E+10	3.43E+10
	Std	8.18E+09	89,561,166	2.71E+10	2.24E+10	2,544,815	9.7E+09	2.34E+10	2.8E+10	1.67E+10	2.85E+08	7,374,891	3.79E+09	8.27E+08	4.35E+09	2.18E+10
	Rank	12	3	13	11	1	10	15	14	9	5	2	8	4	6	7
CEC03	Ave	353,298.7	294,569.3	3,718,006	756,044.1	339,270.6	399,314.7	4.12E+11	15,310,302	276,368.2	643,108.1	1,113,873	854,945.8	544,057.3	390,295.3	953,783.1
	Std	10,495.9	21,142.14	4,128,337	276,199.6	13,893.96	37,294.87	7.97E+11	18,395,921	21,043.15	53,397.2	129,217.1	41,434	155,162	49,504.25	131,015.4
	Rank	4	2	13	9	3	6	15	14	1	8	12	10	7	5	11
CEC04	Ave	101,944.54	1179.5831	96,262.08	92,409.128	1368.321	12,339.293	270,799.61	145,276.92	16,319.20	2359.016	795.9192	12,190.83	1361.444	2088.448	6656.466
	Std	18,309.166	66.4505	11,938.31	13,065.122	122.1895	1195.149	23,881.649	18,696.464	820.030	214.246	63.0896	1673.555	120.840	406.598	1996.070
	Rank	13	2	12	11	4	9	15	14	10	6	1	8	3	5	7
CEC05	Ave	2097.627	1280.933	2360.325	2191.964	945.841	1693.396	3119.092	2459.332	1485.856	1623.203	1249.014	1764.648	1376.699	1010.249	1622.343
	Std	34.239	39.25346	57.647	61.9605	118.542	94.8014	87.2340	114.773	19.236	14.159	43.788	29.640	79.527	44.240	62.798
	Rank	11	4	13	12	1	9	15	14	6	8	3	10	5	2	7
CEC06	Ave	711.111	631.881	728.124	719.281	634.420	679.548	766.976	736.541	678.797	617.688	603.119	667.219	667.610	618.385	672.617
	Std	2.855	4.015	9.051	6.394	6.717	8.726	4.791	3.643	2.993	1.236	0.752	3.028	3.168	4.752	4.218
	Rank	11	4	13	12	5	10	15	14	9	2	1	6	7	3	8
CEC07	Ave	3973.682	1601.900	6784.713	4474.539	1236.418	3421.330	13,605.060	7270.222	3527.810	2051.804	1741.728	4560.807	3136.223	1690.668	3224.124
	Std	90.347	161.715	534.917	521.344	93.781	377.718	568.164	654.671	42.312	37.318	100.206	135.052	205.364	187.352	514.155
	Rank	10	2	13	11	1	8	15	14	9	5	4	12	6	3	7
CEC08	Ave	2596.122	1377.833	2777.239	2627.281	1241.784	1895.857	3574.714	2934.162	2018.710	1898.329	1611.926	2074.589	1835.894	1331.396	1758.926
	Std	25.657	68.975	85.027	61.265	59.469	120.992	46.844	43.659	23.397	43.196	80.188	20.183	49.698	38.782	53.408
	Rank	11	3	13	12	1	7	15	14	9	8	4	10	6	2	5
CEC09	Ave	79,222.24	29,122.33	128,660.1	111,673.2	29,490.81	67,538.21	235,848.2	152,831.8	40,565.62	67,863.99	61,209.63	79,012.54	28,820.27	27,704.02	64,112.62
	Std	3339.864	1659.778	10,524.07	5554.772	17,556.36	10,277.35	10,392.25	11,425.5	1512.182	6235.145	10,585.77	3162.34	1595.513	6824.531	13,595.48
	Rank	11	3	13	12	4	8	15	14	5	9	6	10	2	1	7
CEC10	Ave	32,790.450	19,417.990	35,416.69	33,516.215	18,559.31	32,401.431	36,375.258	34,398.562	20,776.46	29,847.280	15,319.49	32,762.45	16,711.11	28,343.490	19,512.837
	Std	879.312	1047.593	425.501	817.597	7132.740	407.106	440.794	520.312	928.078	389.829	1244.049	259.041	1193.722	4604.623	1175.141
	Rank	11	4	14	12	3	9	15	13	6	8	1	10	2	7	5
CEC11	Ave	284,104	17,053.48	554,277.2	338,236.7	81,392.67	77,729.78	14,809,565	469,379.8	63,239.33	120,986.8	167,235.9	246,033.7	210,863	23,652.52	202,278.1
	Std	34,082.19	5422.174	102,540.6	66,713.98	14,876.86	12,723.94	26,685,425	164,626.9	11,427.57	22,715.01	42,663.2	42,622.43	93,454.82	6154.067	52,814.22
	Rank	11	1	14	12	5	4	15	13	3	6	7	10	9	2	8
CEC12	Ave	1.93E+11	460,434,226	1.78E+11	1.763E+11	4.63E+08	1.42E+10	3.54E+11	1.96E+11	2.42E+10	2.81E+09	1.7E+08	1.07E+10	6.06E+8	1.51E+09	1.978E+10
	Std	1.36E+10	107,771,590	2.67E+10	3.20E+10	1.04E+08	3.17E+09	4.11E+10	2.88E+10	6.97E+09	2.66E+08	1.14E+08	1.04E+09	2.14E+08	2.71E+08	7.336E+09
	Rank	13	2	12	11	3	8	15	14	10	6	1	7	4	5	9
CEC13	Ave	4.58E+10	705,374.6	3.44E+10	3.66E+10	46,143.51	4.69E+08	9.34E+10	4.68E+10	1.52E+09	8,858,153	3,760,681	4,052,036	120,982.1	14,931,836	2.62E+09
	Std	6.47E+09	445,638.9	5.63E+09	3.84E+09	12,587.5	2.73E+08	9.9E+09	6.9E+09	3.52E+08	4,161,014	4,310,239	972,256.9	24,388.88	6,671,448	1.79E+09
	Rank	13	3	11	12	1	8	15	14	9	6	4	5	2	7	10
CEC14	Ave	6.74E+07	2.25E+06	2.02E+08	1.15E+08	2.56E+06	2.58E+06	7.22E+08	3.09E+00	3.52E+06	2.08E+07	1.04E+07	4.44E+07	2.45E+06	2.48E+06	5.86E+06
	Std	4.20E+07	1.12E+06	4.17E+07	6.53E+07	9.72E+05	7.21E+05	2.84E+08	1.09E+08	1.19E+06	6.85E+06	4.49E+06	1.12E+07	1.10E+06	1.27E+06	2.47E+06
	Rank	11	1	13	12	4	5	15	14	6	9	8	10	2	3	7
CEC15	Ave	1.95E+10	9546.696	1.3E+10	1.37E+10	35,189.55	3,470,295	4.82E+10	2.08E+10	3.01E+08	7,078,410	6571.879	15,657.54	38,617.58	754,986.3	5.94E+08
	Std	2.82E+09	2966.846	1.49E+09	2.44E+09	9953.871	3,123,840	6.54E+09	3.87E+09	2.47E+08	2,463,740	2706.805	3315.815	29,318.37	1,050,180	8E+08
	Rank	13	2	11	12	4	7	15	14	9	8	1	3	5	6	10
CEC16	Ave	24,402.47	7196.036	18,935.32	20,688.44	5439.32	8527.542	38,331.94	22,767.99	11,415.94	10,739.32	6311.46	11,300.92	5976.64	5650.51	7333.15
	Std	3534.919	813.892	2167.616	1096.959	410.573	1219.020	7863.731	1145.17	500.003	281.964	511.179	424.847	414.886	470.192	1121.849
	Rank	14	5	11	12	1	7	15	13	10	8	4	9	3	2	6
CEC17	Ave	7,506,495	5203.436	1,696,042	5,354,902	4300.178	6188.381	59,721,631	10,217,110	8101.716	6507.391	5951.282	8219.927	5242.485	4757.864	8528.298
	Std	5,469,303	505.0944	1,208,380	3,019,677	422.1045	545.5352	38,442,244	2,397,608	858.9186	358.4553	597.9158	171.115	501.1035	828.9385	2031.196
	Rank	13	3	11	12	1	6	15	14	8	7	5	9	4	2	10
CEC18	Ave	2.60E+08	3.55E+06	5.02E+08	1.82E+08	1.64E+06	4.07E+06	1.23E+09	5.45E+08	1.57E+06	3.67E+07	1.04E+07	1.31E+08	4.88E+06	4.42E+06	2.41E+07
	Std	8.37E+07	3.89E+05	1.01E+08	5.72E+07	5.30E+05	1.75E+06	3.65E+08	3.26E+08	6.43E+05	1.18E+07	3.79E+06	1.85E+07	1.56E+06	1.50E+06	2.16E+07
	Rank	12	3	13	11	2	4	15	14	1	9	7	10	6	5	8
CEC19	Ave	2.73E+10	1,018,630	1.39E+10	1.38E+10	6,524,024	18,875,435	5.14E+10	1.98E+10	2.66E+08	9,040,765	109,473.1	770,649.9	54,888.12	2,390,721	1.47E+09
	Std	3E+09	542,126.6	3.18E+09	3.84E+09	5,458,763	11,616,502	1.04E+10	2.32E+09	85,076,058	2,358,535	186,753.9	820,071.7	53,159.43	943,459.9	1.37E+09
	Rank	14	4	12	11	6	8	15	13	9	7	2	3	1	5	10
CEC20	Ave	8020.2245	5396.4145	8892.6405	8794.723	4463.43	7233.1513	9916.4206	8897.9015	6600.841	6694.9341	5790.443	7975.430	5644.283	5854.5736	6046.9466
	Std	376.70445	346.05236	299.83103	312.94215	583.2524	381.89934	227.58954	347.79495	174.1747	130.75939	294.7866	257.0606	348.9281	1480.4182	691.95824
	Rank	11	2	13	12	1	9	15	14	7	8	4	10	3	5	6
CEC21	Ave	4888.186	2533.89	4654.448	4606.759	2720.731	3224.877	5524.452	4607.875	3886.961	3456.748	3178.491	3655.453	3364.423	2855.625	3411.429
	Std	141.2145	57.18946	214.5147	198.2536	45.67756	97.90937	272.4738	71.80242	98.18394	13.77816	128.2499	26.63459	108.223	76.3268	208.9475
	Rank	14	1	13	11	2	5	15	12	10	8	4	9	6	3	7
CEC22	Ave	34,490.404	25,105.719	37,645.034	36,570.993	16,826.23	34,600.645	38,334.646	37,449.751	23,964.47	32,419.038	17,160.27	34,504.04	21,157.94	26,213.823	22,792.238
	Std	396.41199	1457.9349	174.97446	474.48635	1091.814	200.1131	587.74333	564.42666	924.0662	762.90312	1744.518	653.493	826.021	7547.8622	1498.8567
	Rank	9	6	14	12	1	11	15	13	5	8	2	10	3	7	4
CEC23	Ave	6503.197	3275.887	5826.441	6630.901	3508.979	3937.316	8480.193	5589.357	8996.312	3788.27	3255.018	4021.647	4101.495	3287.275	4014.837
	Std	315.331	87.22193	384.8876	529.0215	53.98898	111.6973	375.7383	218.6821	245.7108	32.64485	78.62813	18.24601	136.4175	66.19829	183.4196
	Rank	12	2	11	13	4	6	14	10	15	5	1	8	9	3	7
CEC24	Ave	10,331.813	3801.2902	8149.7752	10,531.847	3924.763	5374.5511	14,217.06	8458.3736	7050.088	4404.326	3866.233	4476.147	4803.436	3841.8987	4712.7342
	Std	673.16157	73.831631	636.5428	664.72773	348.1868	168.4099	1940.4872	295.27939	848.3431	19.290105	45.89686	24.21110	164.6322	69.362821	91.601062
	Rank	13	1	11	14	4	9	15	12	10	5	3	6	8	2	7
CEC25	Ave	29,982.11	3766.666	38,265.27	29,475.7	3518.926	10,042.58	141,206.3	64,809.23	9681.781	5455.682	3508.178	18,778.26	3933.946	4654.795	5947.987
	Std	839.0102	46.42058	3295.22	1039.571	81.98765	371.1804	8339.274	8752.447	794.073	253.9241	18.48204	1647.064	82.79425	74.44109	559.7637
	Rank	12	3	13	11	2	9	15	14	8	6	1	10	4	5	7
CEC26	Ave	53,905.554	16,607.433	49,831.049	52,030.564	5346.84	28,800.354	93,352.355	59,272.557	31,138.84	16,865.354	13,607.51	18,747.20	22,075.81	12,563.857	20,319.833
	Std	1261.3088	188.70411	8545.3802	2621.0534	4029.018	2549.202	6546.7066	5126.4462	1337.740	189.60249	79.16086	496.9604	4464.751	1101.7688	2422.8096
	Rank	13	4	11	12	1	9	15	14	10	5	3	6	8	2	7
CEC27	Ave	14,222.25	3200.025	11,482.32	11,104.92	3573.907	4880.305	17,577.51	10,404.1	17,999.45	4620.348	3584.569	4695.712	3776.538	3630.951	3942.932
	Std	951.5254	2.72E-05	593.6496	360.6818	15.45457	87.57426	1018.793	967.0674	804.6664	27.57688	81.8085	127.6579	123.9443	84.29333	129.5346
	Rank	13	1	12	11	2	9	14	10	15	7	3	8	5	4	6
CEC28	Ave	28,955.578	3300.0244	38,716.091	35,506.138	3543.84	12,947.367	70,502.543	470,23.009	14,905.25	7831.266	5528.982	19,013.48	4371.154	5548.1365	11,891.086
	Std	1490.045	0.000134	3428.683	5416.921	54.42149	736.89411	10,771.583	1481.4072	1598.156	595.11732	2352.895	570.0481	196.3864	605.24884	3871.7329
	Rank	11	1	13	12	2	8	15	14	9	6	4	10	3	5	7
CEC29	Ave	584,866.3	6979.847	192,863.7	275,968	7286.966	11,678.47	28,654,765	3,417,453	16,704.44	9806.624	7380.821	11,546.01	7410.963	6868.564	8763.036
	Std	207,043.8	373.0695	80,340.59	244,242.2	422.6287	939.0838	12,289,301	3,286,742	1873.528	236.608	387.5825	305.4215	582.053	349.5731	611.8979
	Rank	13	2	11	12	3	9	15	14	10	7	4	8	5	1	6
CEC30	Ave	3.74E+10	7,082,731.5	3.18E+10	2.936E+10	1.65E+08	575,021,335	7.815E+10	2.79E+10	2.76E+09	12,919,420	60,285.47	3.78E+07	4.13E+06	4.53E+07	9.14E+08
	Std	6.55E+09	3,118,888.5	3.339E+09	4.468E+09	61,999,619	270,105,224	2.44E+10	8.08E+09	1.43E+09	2,453,869.9	42,095.78	1.11E+07	2.95E+06	2.03E+07	1.08E+09
	Rank	14	3	13	12	7	8	15	11	10	4	1	5	2	6	9
Mean Rank		11.8276	2.6552	12.4138	11.6897	2.7241	7.7586	14.9310	13.3103	8.2069	6.6897	3.5517	8.2759	4.6207	3.9310	7.4138
Final Rank		12	1	13	11	2	8	15	14	9	6	3	10	5	4	7

**Table A3:** Wilcoxon signed rank test for all optimization results when *dim* = 50.

Function	COA	KOA	SWO	GMO	OMA	TROA	GO	PSO	DE	SA	ABC	ISSA	IGWO	EWOA
CEC01	6.80E-08	5.99E-07	6.80E-08	6.80E-08	6.80E-08	6.80E-08	6.80E-08	6.80E-08	6.80E-08	6.80E-08	6.73E-08	6.80E-08	9.17E-08	6.80E-08
	-	-	-	-	-	-	-	-	-	+	-	+	-	-
CEC03	6.80E-08	6.16E-07	6.80E-08	6.80E-08	6.80E-08	6.80E-08	6.80E-08	0.69489	6.80E-08	6.80E-08	6.80E-08	6.80E-08	8.29E-05	6.80E-08
	-	-	-	-	-	-	-	=	-	-	-	-	+	-
CEC04	6.80E-08	4.37E-07	6.80E-08	0.00432	6.80E-08	6.80E-08	6.80E-08	6.80E-08	1.06E-07	0.507505	6.80E-08	0.011433	0.239324	6.92E-07
	-	-	-	+	-	-	-	-	-	=	-	=	=	=
CEC05	6.80E-08	0.0000821	6.80E-08	0.00432	6.80E-08	6.80E-08	6.80E-08	6.80E-08	1.06E-07	0.507505	6.80E-08	0.011433	0.239324	6.92E-07
	-	-	-	+	-	-	-	-	-	=	-	-	=	-
CEC06	6.80E-08	7.88E-07	6.80E-08	7.15E-06	0.003638	6.80E-08	6.80E-08	3.79E-06	6.80E-08	6.80E-08	7.38E-06	0.000836	6.80E-08	0.005115
	-	-	-	+	+	-	-	-	+	-	+	-	=	-
CEC07	6.80E-08	3.44E-06	6.80E-08	2.98E-01	2.96E-07	6.80E-08	6.80E-08	7.21E-08	7.58E-06	5.23E-07	2.06E-06	1.20E-06	6.80E-08	3.15E-02
	-	-	-	=	-	-	-	-	+	+	-	-	+	-
CEC08	6.80E-08	2.37E-07	6.80E-08	1.43E-07	0.0023413	6.80E-08	6.80E-08	9.17E-08	0.001481	0.009045	6.80E-08	0.797197	0.000129	0.060111
	-	-	-	+	-	-	-	-	-	=	-	=	+	=
CEC09	6.80E-08	7.23E-08	6.80E-08	5.17E-02	0.635945	6.80E-08	6.80E-08	0.424883	3.07E-06	0.350702	5.87E-06	0.000375	6.80E-08	0.860431
	-	-	-	=	=	-	-	=	-	=	-	-	=	=
CEC10	6.80E-08	0.000766	6.80E-08	0.001625	6.80E-08	6.80E-08	6.80E-08	0.0294409	6.80E-08	1.20E-06	6.80E-08	9.75E-06	0.000836	0.597863
	-	-	-	+	-	-	-	-	-	+	-	+	-	=
CEC11	6.80E-08	6.80E-08	6.80E-08	1.43E-07	6.80E-08	6.80E-08	6.80E-08	6.80E-08	6.80E-08	6.80E-08	6.80E-08	2.96E-07	0.0004155	6.80E-08
	-	-	-	-	-	-	-	-	-	-	-	-	-	-
CEC12	6.80E-08	6.80E-08	6.80E-08	0.297677	7.38E-08	6.80E-08	6.80E-08	6.80E-08	6.80E-08	0.0006868	6.80E-08	2.04E-05	0.560852	6.80E-08
	-	-	-	=	-	-	-	-	-	-	-	+	=	-
CEC13	6.80E-08	3.53E-06	6.80E-08	4.54E-07	3.07E-06	6.80E-08	6.80E-08	6.80E-08	7.90E-08	6.80E-08	1.06E-07	1.06E-07	0.000104	0.067868
	-	-	-	-	-	-	-	-	-	-	+	+	-	=
CEC14	6.80E-08	7.99E-08	6.80E-08	0.101729	0.029441	6.80E-08	6.80E-08	0.0011159	1.38E-06	3.94E-07	2.22E-07	0.198834	0.00556	0.009045
	-	-	-	=	=	-	-	-	-	-	-	=	=	-
CEC15	6.80E-08	1.40E-03	6.80E-08	0.000116	0.003336	6.80E-08	6.80E-08	0.180577	6.80E-08	0.067868	5.17E-06	0.0002	4.54E-07	0.002341
	-	-	-	-	-	-	-	=	-	=	-	+	-	-
CEC16	6.80E-08	6.80E-08	6.80E-08	0.005115	8.60E-06	6.80E-08	6.80E-08	7.90E-08	1.92E-07	0.003336	6.80E-08	0.067868	0.000758	0.000104
	-	-	-	=	-	-	-	-	-	-	-	=	-	-
CEC17	6.80E-08	4.74E-08	6.80E-08	0.090907	2.92E-05	6.80E-08	6.80E-08	1.23E-07	2.30E-05	0.000144	0.0000023	0.027483	0.060111	1.05E-06
	-	-	-	=	-	-	-	-	-	-	-	-	=	-
CEC18	6.80E-08	0.000742	6.80E-08	0.0097864	0.053289	6.80E-08	6.80E-08	0.881731	1.66E-07	0.009786	6.80E-08	0.198834	0.067868	0.273285
	-	-	-	-	=	-	-	=	-	-	-	=	=	=
CEC19	6.80E-08	0.000384	6.80E-08	7.90E-08	0.001359	6.80E-08	6.80E-08	6.80E-08	6.80E-08	0.002561	0.00604	5.90E-02	9.75E-06	6.67E-06
	-	-	-	-	+	-	-	-	-	+	=	=	-	-
CEC20	6.80E-08	4.54E-06	6.80E-08	0.113551	6.80E-08	6.80E-08	6.80E-08	6.80E-08	2.06E-06	0.1135513	6.80E-08	2.96E-07	0.163596	1.80E-06
	-	-	-	=	-	-	-	-	-	-	-	+	=	-
CEC21	6.80E-08	6.80E-08	6.80E-08	6.80E-08	0.542772	6.80E-08	6.80E-08	6.80E-08	3.50E-06	0.336915	6.80E-08	0.394171	7.95E-07	0.0007579
	-	-	-	-	=	-	-	-	-	=	-	=	-	-
CEC22	9.17E-08	4.63E-08	6.80E-08	5.90E-02	0.000375	6.80E-08	6.80E-08	6.80E-08	4.54E-06	5.23E-02	6.80E-08	0.133283	0.053103	0.735268
	-	-	-	=	-	-	-	-	-	=	-	=	=	=
CEC23	6.80E-08	0.000914	6.80E-08	6.80E-08	1.23E-07	6.80E-08	6.80E-08	6.80E-08	0.456951	1.20E-06	5.25E-05	0.616775	7.95E-07	0.002139
	-	-	-	-	-	-	-	-	=	-	-	=	-	-
CEC24	6.80E-08	4.77E-06	6.80E-08	6.17E-02	3.42E-07	6.80E-08	6.80E-08	6.80E-08	6.92E-07	0.218406	2.04E-05	0.009786	0.000375	0.000921
	-	-	-	=	-	-	-	-	-	=	-	-	=	-
CEC25	6.80E-08	3.24E-08	6.80E-08	2.22E-07	0.000735	6.80E-08	6.80E-08	6.80E-08	7.60E-06	9.13E-02	9.21E-05	0.003966	0.542772	0.000305
	-	-	-	+	-	-	-	-	-	=	-	-	=	-
CEC26	6.80E-08	0.000761	6.80E-08	9.17E-08	0.001227	6.80E-08	6.80E-08	6.80E-08	0.218406	0.000104	0.635945	0.261616	3.07E-06	0.755743
	-	-	-	-	-	-	-	-	=	-	=	=	+	=
CEC27	6.80E-08	0.000822	6.80E-08	0.524986	1.92E-07	6.80E-08	6.80E-08	6.80E-08	1.92E-07	0.036048	2.69E-06	0.001349	0.000305	5.90E-05
	-	-	-	=	-	-	-	-	-	-	-	-	-	-
CEC28	6.80E-08	1.87E-06	6.80E-08	1.60E-05	6.80E-08	6.80E-08	6.80E-08	6.80E-08	6.80E-08	6.80E-08	6.80E-08	6.80E-08	6.80E-08	6.80E-08
	-	-	-	-	-	-	-	-	-	-	-	-	-	-
CEC29	6.80E-08	6.74E-08	6.80E-08	0.02748	1.20E-06	6.80E-08	6.80E-08	6.80E-08	0.020735	0.44075	7.90E-08	0.261616	2.96E-07	5.25E-05
	-	-	-	-	-	-	-	-	-	=	-	=	-	-
CEC30	6.80E-08	0.000374	6.80E-08	6.80E-08	6.80E-08	6.80E-08	6.80E-08	6.80E-08	3.47E-08	7.49E-06	6.80E-08	4.54E-06	1.06E-07	0.000129
	-	-	-	-	-	-	-	-	-	-	-	-	-	-
+/=/-	0/0/29	0/0/29	0/0/29	6/10/13	2/4/23	0/0/29	0/0/29	0/4/25	2/2/25	4/10/15	2/2/25	6/11/12	6/11/12	0/7/22

**Table A4:** Wilcoxon signed rank test for all optimization results when *dim* = 100.

Function	COA	KOA	SWO	GMO	OMA	TROA	GO	PSO	DE	SA	ABC	ISSA	IGWO	EWOA
CEC01	6.80E-08	6.80E-08	6.80E-08	6.80E-08	6.80E-08	6.80E-08	6.80E-08	6.80E-08	6.80E-08	6.80E-08	6.80E-08	6.80E-08	6.80E-08	6.80E-08
	-	-	-	+	-	-	-	-	-	+	-	-	-	-
CEC03	6.80E-08	1.93E-05	6.80E-08	5.17E-06	6.80E-08	6.80E-08	6.80E-08	0.0016253	6.80E-08	6.80E-08	6.80E-08	1.66E-07	1.43E-07	6.80E-08
	-	-	-	-	-	-	-	+	-	-	-	-	-	-
CEC04	6.80E-08	4.52E-06	6.80E-08	6.80E-08	6.80E-08	6.80E-08	6.80E-08	6.80E-08	6.80E-08	6.80E-08	6.80E-08	0.0013486	6.80E-08	6.80E-08
	-	-	-	-	-	-	-	-	-	+	-	-	-	-
CEC05	6.80E-08	6.80E-08	6.80E-08	6.80E-08	0.4248835	6.80E-08	6.80E-08	0.001014	0.009786	6.90E-02	6.80E-08	3.99E-06	6.80E-08	0.063892
	-	-	-	-	=	-	-	-	-	=	-	-	+	=
CEC06	6.80E-08	4.64E-06	6.80E-08	9.31E-06	0.0027986	6.80E-08	6.80E-08	0.0003048	6.80E-08	9.80E-01	0.001227	0.016669	6.80E-08	0.903116
	-	-	-	-	-	-	-	-	+	=	-	-	+	=
CEC07	6.80E-08	6.80E-08	6.80E-08	6.80E-08	3.74E-06	6.80E-08	6.80E-08	6.80E-08	0.0012941	7.90E-08	6.80E-08	3.50E-06	1.66E-07	6.80E-08
	-	-	-	+	-	-	-	-	-	-	-	-	-	-
CEC08	6.80E-08	3.78E-06	6.80E-08	3.94E-01	0.9031165	6.80E-08	6.80E-08	0.000222	0.903116	2.22E-07	3.94E-07	0.049864	3.42E-07	0.015479
	-	-	-	=	=	-	-	-	=	-	-	-	+	-
CEC09	6.80E-08	7.91E-05	6.80E-08	1.81E-05	0.0047025	6.80E-08	6.80E-08	6.80E-08	0.003966	0.989209	6.01E-07	2.96E-07	6.80E-08	0.285305
	-	-	-	-	-	-	-	-	-	=	-	+	+	=
CEC10	6.80E-08	6.80E-08	6.80E-08	1.20E-06	6.80E-08	6.80E-08	6.80E-08	0.000921	6.80E-08	6.80E-08	6.80E-08	1.80E-06	0.119856	0.013321
	-	-	-	+	-	-	-	-	-	+	-	+	=	-
CEC11	6.80E-08	8.37E-06	6.80E-08	6.80E-08	7.90E-08	6.80E-08	6.80E-08	6.80E-08	6.80E-08	9.17E-08	6.80E-08	6.80E-08	0.027483	6.80E-08
	-	-	-	-	-	-	-	-	-	-	-	-	-	-
CEC12	6.80E-08	9.17E-08	6.80E-08	0.394171	6.80E-08	6.80E-08	6.80E-08	6.80E-08	6.80E-08	7.95E-07	6.80E-08	0.989209	6.80E-08	6.80E-08
	-	-	-	=	-	-	-	-	-	=	-	=	-	-
CEC13	6.80E-08	7.81E-09	6.80E-08	8.59E-02	6.80E-08	6.80E-08	6.80E-08	6.80E-08	1.23E-07	0.655361	4.54E-06	6.01E-07	6.92E-07	0.000247
	-	-	-	=	-	-	-	-	-	=	-	+	-	-
CEC14	6.80E-08	5.56E-06	6.80E-08	0.085855	0.490334	6.80E-08	6.80E-08	0.946084	6.80E-08	9.17E-08	6.80E-08	0.119856	0.261616	1.60E-05
	-	-	-	=	=	-	-	=	-	-	-	=	=	-
CEC15	6.80E-08	6.80E-08	6.80E-08	3.94E-07	6.80E-08	6.80E-08	6.80E-08	6.80E-08	6.80E-08	0.0810312	5.90E-05	4.54E-07	6.80E-08	1.25E-05
	-	-	-	-	-	-	-	-	-	=	-	-	-	-
CEC16	6.80E-08	8.97E-06	6.80E-08	5.59E-02	4.68E-05	6.80E-08	6.80E-08	6.80E-08	6.80E-08	0.033718	6.80E-08	1.81E-05	4.54E-06	0.014364
	-	-	-	=	-	-	-	-	-	=	-	+	+	-
CEC17	6.80E-08	0.006423	6.80E-08	1.05E-06	0.597863	6.80E-08	6.80E-08	2.22E-07	6.80E-08	0.946084	6.80E-08	0.694891	5.87E-06	3.50E-06
	-	-	-	-	=	-	-	-	-	=	-	=	+	-
CEC18	6.80E-08	6.80E-08	6.80E-08	0.000416	0.560852	6.80E-08	6.80E-08	2.04E-05	6.80E-08	2.22E-07	6.80E-08	0.033718	0.597863	1.23E-07
	-	-	-	=	=	-	-	+	-	-	-	-	=	-
CEC19	6.80E-08	5.91E-08	6.80E-08	4.68E-05	6.80E-08	6.80E-08	6.80E-08	6.80E-08	4.13E-05	3.94E-07	1.38E-06	1.06E-07	1.41E-05	4.68E-05
	-	-	-	-	-	-	-	-	-	+	+	+	-	-
CEC20	6.80E-08	1.31E-07	6.80E-08	0.001227	6.80E-08	6.80E-08	6.80E-08	6.80E-08	6.80E-08	0.119856	6.80E-08	0.379332	0.02227	4.17E-05
	-	-	-	+	-	-	-	-	-	=	-	=	-	-
CEC21	6.80E-08	7.93E-07	6.80E-08	6.80E-08	0.002799	6.80E-08	6.80E-08	6.80E-08	0.597863	1.38E-06	2.06E-06	0.228694	6.80E-08	0.323482
	-	-	-	-	-	-	-	-	=	-	-	=	-	-
CEC22	9.17E-08	6.32E-06	6.80E-08	1.80E-06	6.80E-08	6.80E-08	6.80E-08	0.0097865	6.80E-08	6.80E-08	6.80E-08	1.06E-07	1.81E-05	3.07E-06
	-	-	-	+	-	-	-	+	-	+	-	+	-	+
CEC23	6.80E-08	3.86E-07	6.80E-08	3.94E-07	0.000144	6.80E-08	6.80E-08	6.80E-08	0.473481	6.80E-08	7.58E-06	0.033718	7.90E-08	1.41E-05
	-	-	-	-	-	-	-	-	=	+	-	-	-	-
CEC24	6.80E-08	0.000037	6.80E-08	6.80E-08	6.80E-08	6.80E-08	6.80E-08	6.80E-08	6.01E-07	6.80E-08	8.29E-05	0.261616	7.90E-08	0.090907
	-	-	-	-	-	-	-	-	-	-	-	=	-	=
CEC25	6.80E-08	9.87E-06	6.80E-08	1.23E-07	6.80E-08	6.80E-08	6.80E-08	6.80E-08	6.80E-08	5.23E-01	6.80E-08	2.36E-06	6.80E-08	6.80E-08
	-	-	-	+	-	-	-	-	-	=	-	-	-	-
CEC26	6.80E-08	6.80E-08	6.80E-08	5.16E-02	9.17E-08	6.80E-08	6.80E-08	6.80E-08	3.99E-06	6.80E-08	0.038515	0.776391	6.80E-08	0.180577
	-	-	-	=	-	-	-	-	-	-	-	=	=	=
CEC27	6.80E-08	6.80E-08	6.80E-08	9.13E-07	1.43E-07	6.80E-08	6.80E-08	6.80E-08	2.30E-05	6.01E-07	3.50E-06	1.25E-05	9.13E-07	0.04986369
	-	-	-	-	-	-	-	-	-	-	-	-	-	=
CEC28	6.80E-08	6.80E-08	6.80E-08	6.80E-08	6.80E-08	6.80E-08	6.80E-08	6.80E-08	6.80E-08	6.80E-08	6.80E-08	6.80E-08	6.80E-08	6.80E-08
	-	-	-	-	-	-	-	-	-	-	-	-	-	-
CEC29	6.80E-08	6.80E-08	6.80E-08	1.66E-07	2.22E-07	6.80E-08	6.80E-08	6.80E-08	2.92E-05	6.80E-08	6.80E-08	4.54E-07	6.01E-01	0.0565165
	-	-	-	-	-	-	-	-	-	-	-	-	=	=
CEC30	6.80E-08	6.80E-08	6.80E-08	6.80E-08	6.80E-08	6.80E-08	6.80E-08	6.80E-08	0.085855	6.80E-08	3.42E-07	6.80E-08	1.92E-07	0.002139
	-	-	-	-	-	-	-	-	=	-	-	=	-	-
+/=/-	0/0/29	0/0/29	0/0/29	6/7/16	0/5/24	0/0/29	0/0/29	3/1/25	1/4/24	6/10/14	1/0/28	6/8/14	6/5/18	1/7/21
